# Supplementary material for: Identification of Freezing-Responsive microRNAs and Their Targets in Chinese Jujube by Small RNA and Degradome Sequencing
Source: Int J Mol Sci. 2026 Sep 4;27(17):7912. doi: 10.3390/ijms27177912 (PMC13566890; doi:10.3390/ijms27177912)
Supplement: Supplementary file 1 [file ijms-27-07912-s001.zip › Supplementary Material S2/novel_miR_174.pdf]

Provisional ID : novel_miR_174
Score total : 71764.6
Score for star read(s) : 3.9
Score for read counts : 71758
Score for mfe : 1
Score for randfold : 1.6
Score for cons. seed :
Total read count : 140762
Mature read count : 140253
Loop read count : 1
Star read count : 508

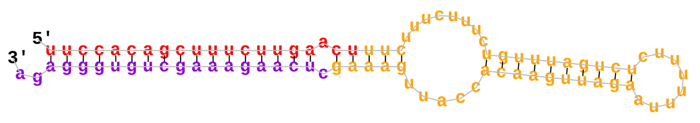

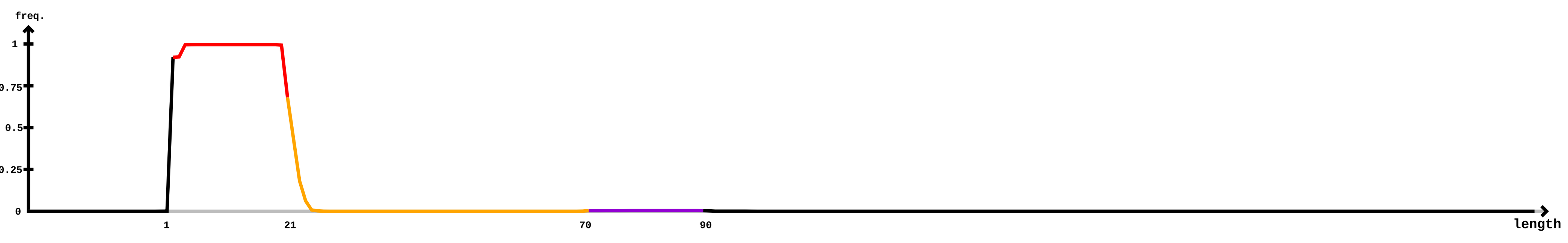

Mature Star

```
5'- uuugcaaguccuggucaugcuuuuccacagcuuucuugaacuuucuuucuuucuguuuagucucuuuuuuaagauugaacaccauugaaagcucaagaaagcugugggagaauauggcaaauuugggauuuuaauuuuuuuauuuuuguuuuuauuuuuuauuuuuccuucaacuacucuucagcugaugaugauguugcacacagaaagaaagaaagaaagaaagaaagagagaucucagaagcuaaau -3' obs
    uuugcaaguccuggucaugcuuuuccacagcuuucuugaacuuucuuucuuucuguuuagucucuuuuuuaagauugaacaccauugaaagcucaagaaagcugugggagaauauggcaaauuugggauuuuaauuuuuuuauuuuuguuuuuauuuuuuauuuuuccuucaacuacucuucagcugaugaugauguugcacacagaaagaaagaaagaaagaaagaaagagagaucucagaagcuaaau     exp
    ............(((((.((((((((((((((((((.(((((........(((((((((........)))))))))).....))))).))))))))))))))))))).))))))...((((((((((..(((((((.(((((.(((((.((((((..........((((...((.(((.....))).)).))))......)))))).))))).))))).))))))))))))))))).......  reads  mm  sample
    ....................uuuuccacagcuuucuugaac.........................................................................................................................................................................................................  7  0  JCK-3
    ....................uuuuccacagcuuucuugaac.........................................................................................................................................................................................................  3  0  LT-3
    ....................uuuuccacagcuuucuugaac.........................................................................................................................................................................................................  2  0  JT-2
    ....................uuuuccacagcuuucuugaac.........................................................................................................................................................................................................  3  0  LT-1
    ....................uuuuccacagcuuucuugaac.........................................................................................................................................................................................................  3  0  LCK-3
    ....................uuuuccacagcuuucuugaac.........................................................................................................................................................................................................  4  0  JCK-1
    ....................uuuuccacagcuuucuugaac.........................................................................................................................................................................................................  3  0  JT-3
    ....................uuuuccacagcuuucuugaac.........................................................................................................................................................................................................  6  0  JCK-2
    ....................uuuuccacagcuuucuugaacu........................................................................................................................................................................................................  1  0  JCK-3
    ....................uuuuccacagcuuucuugaacu........................................................................................................................................................................................................  4  0  JCK-2
    ....................uNuuccacagcuuucuugaacu........................................................................................................................................................................................................  1  1  JCK-3
    .....................uuuccacagcuuucuuga...........................................................................................................................................................................................................  2  0  JCK-1
    .....................Auuccacagcuuucuugaacu........................................................................................................................................................................................................  1  1  JCK-3
    .....................uuuccacagcuuucuugaacu........................................................................................................................................................................................................  1  0  JCK-2
    .....................uuuccacagcuuucuugaacu........................................................................................................................................................................................................  1  0  LT-1
    .....................uuuccacagcuuucuugaacu........................................................................................................................................................................................................  2  0  LCK-3
    .....................uuuccacagcuuucuugaacu........................................................................................................................................................................................................  1  0  JCK-3
    .....................Auuccacagcuuucuugaacu........................................................................................................................................................................................................  2  1  JCK-2
    .....................Guuccacagcuuucuugaacu........................................................................................................................................................................................................  2  1  LT-3
    .....................Cuuccacagcuuucuugaacuu.......................................................................................................................................................................................................  3  1  LT-3
    .....................Cuuccacagcuuucuugaacuu.......................................................................................................................................................................................................  1  1  LT-2
    .....................Cuuccacagcuuucuugaacuu.......................................................................................................................................................................................................  3  1  LT-1
    .....................Cuuccacagcuuucuugaacuu.......................................................................................................................................................................................................  3  1  JCK-2
    .....................Cuuccacagcuuucuugaacuu.......................................................................................................................................................................................................  2  1  JCK-1
    .....................Cuuccacagcuuucuugaacuuu......................................................................................................................................................................................................  1  1  LT-3
    .....................Cuuccacagcuuucuugaacuuu......................................................................................................................................................................................................  1  1  LT-1
    .....................Cuuccacagcuuucuugaacuuu......................................................................................................................................................................................................  2  1  JCK-1
    .....................Cuuccacagcuuucuugaacuuu......................................................................................................................................................................................................  3  1  LCK-3
    ......................uuccacagcuuucuugaC...........................................................................................................................................................................................................  1  1  JCK-3
    ......................uuccacagcuuucuugCa...........................................................................................................................................................................................................  1  1  LT-2
    ......................uuccacagcuuucuugaC...........................................................................................................................................................................................................  1  1  LT-3
    ......................uuccacagcuuucuugaa...........................................................................................................................................................................................................  31  0  LT-1
    ......................uuccacagcuuucuugaa...........................................................................................................................................................................................................  46  0  LCK-1
```

Mature **Star**

uuugcaaguccuggucaugcuuuuccacagcuuucuugaacuuucuuucuuucuguuuagucucuuuuuuaagauugaacaccauugaaagcucaagaaagcugugggagaauauggcaaauuugggauuuuaauuuuuuuauuuuuguuuuuauuuuuuauuuuuccuucaacuacucuucagcugaugaugauguugcacacagaaagaaagaaagaaagaaagaaagagagaucucagaagcuaaau

uuccacagcuuucuugaa	53	0	LCK-2
uuccacagcuuucuugUa	5	1	JT-1
uuccacagcuuucuugaC	4	1	LT-2
uuccacagcuuucuugUa	2	1	JCK-3
uuccacagcuuucuugaa	43	0	LCK-3
uuccacagcuuucuugaa	9	0	JCK-3
uuccacagcuuucuugUa	1	1	LT-1
uuccacagcuuucuuUaa	1	1	LT-1
uuccacagcuuucuugaU	3	1	LT-2
uuccaNagcuuucuugaa	1	1	LCK-1
uuccacagcuuucuugUa	4	1	LT-3
uuccacagcuuucuugaa	52	0	LT-3
uuccacagcuuucuugaU	11	1	LT-1
uuccacagcuuucuugUa	15	1	LT-2
uuccacagcuuucuugaU	1	1	JCK-1
uuccacagcuuucuugUa	19	1	LCK-3
uuccacagcuuucuugaU	4	1	LCK-3
uuccacagcuuucuugaU	1	1	JCK-2
uuccacagcuuucuugaa	15	0	JT-3
uuccacagcuuucuugaa	5	0	JT-2
uuccacagcuuucuugaa	43	0	LT-2
uuccacagcuuucuGgaa	1	1	LCK-1
uuccacagcuuucuugaU	14	1	LT-3
uuccacagcuuucuugaa	16	0	JCK-1
uuccacagcuuucuugaa	15	0	JCK-2
uuccacagcuuucuugGa	1	1	LCK-2
uuccacagcuGucuugaac	1	1	LT-1
uuccaAagcuuucuugaac	1	1	LT-2
uuccacagcuuucGugaac	3	1	LCK-1
uuccacagcCuucuugaac	1	1	JCK-1
uuccacaAcuuucuugaac	1	1	JCK-3
uuccacagcuuucuugaaU	98	1	LT-3
uuccacagcuuucuugaCc	7	1	JCK-2
uuccCcagcuuucuugaac	1	1	LCK-2
uuccacGgcuuucuugaac	2	1	JCK-2
uNccacagcuuucuugaac	3	1	JT-1
uuccacagcuuCcuugaac	3	1	JCK-1
uuccacCgcuuucuugaac	2	1	JT-1
uuccacagcuuuUuugaac	8	1	JCK-2
uucAacagcuuucuugaac	1	1	LT-2
uuccacCgcuuucuugaac	2	1	JCK-3
uuccacagcCuucuugaac	7	1	LT-1
uuccacagcuuucuugaac	8638	0	LT-3
uuccCcagcuuucuugaac	13	1	LCK-3
uuccacagcuuucuugaUc	3	1	LT-2
uuccCcagcuuucuugaac	2	1	JCK-3
uuccacagcCuucuugaac	1	1	JCK-2
uNccacagcuuucuugaac	1	1	LCK-2
uuccacagcuuucuCgaac	1	1	LT-1
uuccacagcuuucuugaac	8415	0	LCK-3
uuccacaAcuuucuugaac	1	1	JCK-2
uuccacagcuuucuugCac	1	1	LCK-1
uuccacagcuuuUuugaac	1	1	LCK-2
uuccacagcuuucuugaCc	1	1	LCK-1
uuUcacagcuuucuugaac	1	1	JCK-3
uuccacagcuuucuugUac	3	1	LCK-3
uCccacagcuuucuugaac	2	1	LT-3
uuccacagcuuucuugaaU	28	1	JCK-3
uuccaUagcuuucuugaac	2	1	LT-3
uuccacagcuuucuugGac	1	1	JCK-3
uuccacagcuuucuuAaac	1	1	JCK-1
uuccacagcuuucuugCac	4	1	LT-1
Cuccacagcuuucuugaac	1	1	JCK-3
uuccacagcuuucuugaac	2180	0	JCK-1
uuccacagUuuucuugaac	1	1	LT-3
uuccacagcuuCcuugaac	3	1	LCK-3
uuccacagcuuucuugaUc	2	1	JCK-1
uuccaUagcuuucuugaac	1	1	JCK-2
uuccacCgcuuucuugaac	12	1	LT-1
Cuccacagcuuucuugaac	1	1	LT-2

Mature **Star**

uugcaaguccuggucaugcuuuuccacagcuuucuugaacuuucuuucuuucuguuuagucucuuuuuuaagauugaacaccauugaaagcucaagaaagcugugggagaauauggcaaauuugggauuuuaauuuuuuuauuuuuguuuuuauuuuuuauuuuuccuucaacuacucuucagcugaugaugauguugcacacagaaagaaagaaagaaagaaagaaagagagaucucagaagcuaaau			
uuccacagUuucuugaac	5	1	LT-1
uuccacagcuuucuugaaU	24	1	LCK-2
uuccacagcuCucuugaac	2	1	JCK-2
uuccacagcuuucuugaac	542	0	JT-3
uuccCcagcuuucuugaac	16	1	LT-3
uuccacagcuuuAuugaac	1	1	LT-2
uuccacaAcuuucuugaac	2	1	LT-2
uuccCcagcuuucuugaac	12	1	LT-1
uuccacagcuuucuugaUc	2	1	LT-3
uuccacagcuuucuugaUc	3	1	JT-1
uuccacagcuuucuugaaU	53	1	JCK-2
uuccacaAcuuucuugaac	5	1	LT-1
uuccacagcuuucuugaGc	1	1	JCK-1
uuccacagcuuNcuugaac	2	1	JCK-1
uucNacagcuuucuugaac	1	1	JCK-1
uuccacagcuuucuugaac	6061	0	LT-1
uuUcacagcuuucuugaac	1	1	LT-1
uuccaAagcuuucuugaac	3	1	LT-3
uuccacagcuuucuuUaac	1	1	JT-1
uuccCcagcuuucuugaac	4	1	JCK-2
uuNcacagcuuucuugaac	1	1	LCK-1
uuccacaAcuuucuugaac	1	1	LT-3
uuccacagcuuucuugaac	1676	0	LCK-1
uuUcacagcuuucuugaac	2	1	JCK-2
uuccacagcuuucuNgaac	4	1	LCK-1
uuccacagcuuucuGgaac	2	1	LCK-1
uuccacagcuuucNugaac	6	1	LCK-1
uCccacagcuuucuugaac	1	1	JCK-1
uuccacagcuuucuugaaU	24	1	JCK-1
uuccacagcuuucCugaac	1	1	JCK-3
uuccacagcuuucuugaGc	1	1	LCK-3
uuccacagcuuucuugCac	1	1	LCK-2
uuccacagcuuuUugaac	2	1	JCK-3
uuccacagcuuucuugCac	11	1	LT-3
uuccacagcuuucuGgaac	1	1	JCK-2
uuccacagcuuucuugaaU	103	1	LCK-3
uuccacagcuuucCugaac	2	1	LT-1
uuccacagUuucuugaac	3	1	LT-2
uuccacagcuuucuugaCc	13	1	LT-2
uuccacagcCuucuugaac	5	1	LT-3
uuccacCgcuuucuugaac	2	1	LCK-2
uuccaUagcuuucuugaac	1	1	LT-2
uuccacagcuuCcuugaac	3	1	JCK-2
uCccacagcuuucuugaac	1	1	JCK-2
uuccacagcuuucCugaac	2	1	LCK-3
uuccacaAcuuucuugaac	2	1	LCK-3
uuccacagcuuucuugaac	1423	0	JT-1
uuccacagUuucuugaac	4	1	LCK-3
uuccacagcuuucuugaCc	8	1	LCK-3
uNccacagcuuucuugaac	1	1	JCK-1
uuccCcagcuuucuugaac	2	1	JT-1
uuccacagcCuucuugaac	1	1	LT-2
uuUcacagcuuucuugaac	6	1	JCK-1
uuccacagcuuucuuAaac	4	1	LT-2
uuccacagcuuucuugCac	5	1	LT-2
uuccacagcuuucuugaCc	2	1	LCK-2
uuccacagcuuucuugaac	5694	0	LT-2
uuAcacagcuuucuugaac	1	1	LT-3
uNccacagcuuucuugaac	5	1	JCK-2
uuccacagcuuucuugaac	1739	0	LCK-2
uNccacagcuuucuugaac	2	1	JCK-3
uuccaAagcuuucuugaac	1	1	JCK-2
uuccacagcuuucuugaUc	10	1	LCK-3
uuccacagcuuucuugaCc	5	1	LT-1
uuccCcagcuuucuugaac	33	1	LT-2
uuccGcagcuuucuugaac	1	1	LT-1
Cuccacagcuuucuugaac	2	1	JT-1
uuccaNagcuuucuugaac	3	1	LCK-1
uuccacagAuuucuugaac	1	1	LT-2
uucUacagcuuucuugaac	1	1	JCK-3

Mature **Star**

uuugcaaguccuggucaugcuuuuccacagcuuucuugaacuuucuuucuuucuguuuagucucuuuuuuaagauugaacaccauugaaagcucaagaaagcugugggagaauauggcaaauuugggauuuuaauuuuuuuauuuuuguuuuuauuuuuuauuuuuccuucaacuacucuucagcugaugaugauguugcacacagaaagaaagaaagaaagaaagaaagagagaucucagaagcuaaau			
uuccCcagcuuucuugaac	1	1	JCK-1
uuccacagcuuCcuugaac	2	1	LT-1
uuccacagcuuucuugCac	3	1	LCK-3
uuGcacagcuuucuugaac	3	1	LT-2
uuccacCgcuuucuugaac	9	1	JCK-2
uuccacagcuuucuugCac	3	1	JT-1
uuccacagcuuucuugaac	3571	0	JCK-2
uuccacagcuuucCugaac	1	1	LT-2
uuNcacagcuuucuugaac	14	1	LT-2
uuccacagcuuuNuugaac	1	1	LCK-1
uuccacagcuuucuugCac	2	1	JCK-1
Cuccacagcuuucuugaac	3	1	LT-3
uuccCcagcuuucuugaac	1	1	JT-3
uuccacagcuuucuugaCc	1	1	JCK-3
uuccacCgcuuucuugaac	2	1	JCK-1
uuccacagcuuuUuugaac	3	1	LT-1
uuccacagcuuucuugaCc	1	1	JT-1
uuccacagcuGucuugaac	3	1	JCK-1
uuccacaUcuuucuugaac	2	1	JCK-2
uuccacagcuuuUuugaac	6	1	LT-2
Cuccacagcuuucuugaac	4	1	LT-1
uuccacagcuuuAuugaac	2	1	LCK-3
uuccacagcuuucCugaac	3	1	JCK-2
uuccacagcuuucuugaaU	64	1	LT-2
uuccacCgcuuucuugaac	11	1	LCK-3
uuccaAagcuuucuugaac	1	1	JCK-1
uuccacagcuuucuugUac	1	1	LT-3
uuccaAagcuuucuugaac	1	1	LCK-3
uucUacagcuuucuugaac	2	1	JCK-1
uuccacagcuuucuugaCc	10	1	LT-3
uuccacagcuuucuugaaU	26	1	LCK-1
uuccacagcuuuUuugaac	4	1	JCK-1
uucUacagcuuucuugaac	4	1	JCK-2
uCccacagcuuucuugaac	1	1	LCK-1
uuccacagcuuucuugCac	5	1	JCK-2
uuccacagcuuucuugaGc	1	1	LT-3
uuccacagcuuucuugaac	2667	0	JCK-3
uuccacagcuuuAuugaac	2	1	LT-1
uuccacCgcuuucuugaac	16	1	LT-3
uuccacagcuuucuugaaU	37	1	LT-1
uuccacCgcuuucuugaac	1	1	JT-2
uuccacagcuCucuugaac	1	1	LT-1
uuAcacagcuuucuugaac	1	1	JCK-1
uuUcacagcuuucuugaac	1	1	LT-3
uuccacagcuuCcuugaac	2	1	JCK-3
Cuccacagcuuucuugaac	3	1	LCK-3
uuccacagcuuucuugCac	1	1	JCK-3
uuccacCgcuuucuugaac	10	1	LT-2
uuccCcagcuuucuugaac	2	1	LCK-1
uuccacagcuuucuugaac	411	0	JT-2
uuccacagcuCucuugaacu	1	1	JT-1
uuccacagcuuucuugCacu	2	1	JCK-3
uuccacagcuuNcuugaacu	3	1	JT-1
uuccacagcuuucuugaCcu	1	1	JCK-3
uuccacagcuuucuugaCcu	11	1	LCK-3
uuccacagcuuucuugCacu	2	1	LCK-2
uuccacagcuuucNugaacu	5	1	LCK-1
uCccacagcuuucuugaacu	1	1	LT-3
uuccaAagcuuucuugaacu	2	1	JCK-1
uuccacagcuuucuugaCcu	1	1	JT-2
uCccacagcuuucuugaacu	3	1	JCK-3
Cuccacagcuuucuugaacu	1	1	LT-3
uuccacagcuuucuugaacC	18	1	LT-3
uuccacagcCuucuugaacu	1	1	JCK-1
uuccacagcuuucuugCacu	10	1	LT-2
uuccacCgcuuucuugaacu	9	1	LCK-3
uuccacagcuuucuugGacu	1	1	LT-2
uuccacagcuuucuugaaUu	24	1	LCK-1
uuccacagcuuucuugCacu	6	1	LT-3
uuccacagcuuNcuugaacu	1	1	JT-2

```
                      Mature                                                                     Star
uuugcaaguccuggucaugcuuuuccacagcuuucuugaacuuucuuucuuucuguuuagucucuuuuuuaagauugaacaccauugaaagcucaagaaagcugugggagaauauggcaaauuugggauuuuaauuuuuuuauuuuuguuuuuauuuuuuauuuuuccuucaacuacucuucagcugaugaugauguugcacacagaaagaaagaaagaaagaaagaaagagagaucucagaagcuaaau
......................uNccacagcuuucuugaacu................................................................................................................................................................................................   1     1   JT-1
......................uuccacagcuuucuugaacG................................................................................................................................................................................................   2     1   JCK-2
......................uuccacagcuuucuugaacA................................................................................................................................................................................................   2     1   LCK-1
......................uuccacagcuuucuCgaacu................................................................................................................................................................................................   1     1   LT-3
......................uuccCcagcuuucuugaacu................................................................................................................................................................................................   1     1   LCK-1
......................uuccaAagcuuucuugaacu................................................................................................................................................................................................   1     1   JCK-2
......................uuccacagcuuucCugaacu................................................................................................................................................................................................   1     1   LT-3
......................Cuccacagcuuucuugaacu................................................................................................................................................................................................   2     1   JCK-3
......................uucNacagcuuucuugaacu................................................................................................................................................................................................   2     1   JCK-1
......................uCccacagcuuucuugaacu................................................................................................................................................................................................   2     1   JCK-1
......................uuccacagcuuucuugaacA................................................................................................................................................................................................   3     1   JT-2
......................uuccacagcuuucuCgaacu................................................................................................................................................................................................   1     1   LT-2
......................uuccacagcuuucuugaaUu................................................................................................................................................................................................   45    1   JT-2
......................uuccacagcuuucuugCacu................................................................................................................................................................................................   2     1   LCK-1
......................uuccacagcuuucuugCacu................................................................................................................................................................................................   3     1   JCK-2
......................uuccCcagcuuucuugaacu................................................................................................................................................................................................   8     1   LCK-3
......................uuccacagcuuucCugaacu................................................................................................................................................................................................   1     1   LT-1
......................uuccacagcuuucCugaacu................................................................................................................................................................................................   2     1   LCK-3
......................uuccacagcuuucuugaCcu................................................................................................................................................................................................   3     1   JCK-2
......................uuccacagcuuucuugaCcu................................................................................................................................................................................................   1     1   LCK-2
......................uNccacagcuuucuugaacu................................................................................................................................................................................................   1     1   JCK-2
......................uuccUcagcuuucuugaacu................................................................................................................................................................................................   1     1   JCK-2
......................uuccacagcuuucuuCaacu................................................................................................................................................................................................   1     1   JCK-1
......................uuccacagcuuucuugaacG................................................................................................................................................................................................   6     1   LT-3
......................uuccacagUuuucuugaacu................................................................................................................................................................................................   1     1   LCK-3
......................uuccaUagcuuucuugaacu................................................................................................................................................................................................   3     1   JCK-2
......................uuccacagcuuucuugaacC................................................................................................................................................................................................   8     1   JT-1
......................uuccacagcuuucuuUaacu................................................................................................................................................................................................   1     1   LT-2
......................uuccCcagcuuucuugaacu................................................................................................................................................................................................   2     1   JT-3
......................uuccCcagcuuucuugaacu................................................................................................................................................................................................   3     1   LT-1
......................uuccacagcuuucuuAaacu................................................................................................................................................................................................   1     1   JCK-1
......................uuccacagcuuucuugaacA................................................................................................................................................................................................   43    1   JCK-3
......................uuccacagcuuucuugaacA................................................................................................................................................................................................   20    1   JT-1
......................uuccacagcCuucuugaacu................................................................................................................................................................................................   1     1   JCK-3
......................uuccacCgcuuucuugaacu................................................................................................................................................................................................   2     1   LCK-1
......................uuccacagcuuucCugaacu................................................................................................................................................................................................   1     1   JCK-2
......................uNccacagcuuucuugaacu................................................................................................................................................................................................   2     1   LCK-1
......................uuccacaAcuuucuugaacu................................................................................................................................................................................................   4     1   LCK-3
......................uCccacagcuuucuugaacu................................................................................................................................................................................................   1     1   LCK-3
......................uuccacagcuuucuugaaUu................................................................................................................................................................................................   111   1   JCK-2
......................uuccacCgcuuucuugaacu................................................................................................................................................................................................   4     1   LT-1
......................uuccacagcuuucuNgaacu................................................................................................................................................................................................   7     1   LCK-1
......................uuccacagcCuucuugaacu................................................................................................................................................................................................   1     1   JT-3
......................uuccacagcuuucuugaacC................................................................................................................................................................................................   9     1   LT-1
......................uuccacagcuuucuugaCcu................................................................................................................................................................................................   8     1   LT-3
......................uuccaAagcuuucuugaacu................................................................................................................................................................................................   3     1   LT-1
......................uuNcacagcuuucuugaacu................................................................................................................................................................................................   33    1   LT-2
......................uuccacagcCuucuugaacu................................................................................................................................................................................................   5     1   LT-1
......................uuccacagcuuucuugaacu................................................................................................................................................................................................   4415  0   LT-1
......................uuccacagcuuucuugaaAu................................................................................................................................................................................................   6     1   LT-2
......................uuccacagcuuucuugaacC................................................................................................................................................................................................   41    1   LCK-3
......................uuccacagcCuucuugaacu................................................................................................................................................................................................   7     1   LT-2
......................uuccacagcuuucuugaacu................................................................................................................................................................................................   2326  0   LCK-2
......................uuccacagcuGucuugaacu................................................................................................................................................................................................   1     1   JCK-1
......................uuccacagcuuucuugaacG................................................................................................................................................................................................   2     1   LT-1
......................uuccacagcuuucuugaacA................................................................................................................................................................................................   172   1   LT-3
......................uuccacagcuGucuugaacu................................................................................................................................................................................................   1     1   LT-2
......................Cuccacagcuuucuugaacu................................................................................................................................................................................................   2     1   LCK-1
......................uuccCcagcuuucuugaacu................................................................................................................................................................................................   8     1   LT-3
......................uNccacagcuuucuugaacu................................................................................................................................................................................................   4     1   JCK-1
......................uuccacagcuuucuugaGcu................................................................................................................................................................................................   3     1   JCK-1
......................uuccacagcuuucuugaacG................................................................................................................................................................................................   1     1   JT-1
......................uuccacaAcuuucuugaacu................................................................................................................................................................................................   1     1   LCK-2
......................uuccacCgcuuucuugaacu................................................................................................................................................................................................   8     1   LT-3
......................uuccacCgcuuucuugaacu................................................................................................................................................................................................   13    1   LT-2
......................uuccacagcuuucuugaacN................................................................................................................................................................................................   2     1   JCK-1
......................uuccacagcuuuUuugaacu................................................................................................................................................................................................   1     1   LT-3
......................uuccacagcuuucuugaaUu................................................................................................................................................................................................   192   1   LCK-3
......................uuccUcagcuuucuugaacu................................................................................................................................................................................................   1     1   JCK-1
......................uuccacagcuuucuugaaAu................................................................................................................................................................................................   1     1   JCK-1
```

```
                      Mature                                                                 Star
uuugcaaguccuggucaugcuuuuccacagcuuucuugaacuuucuuucuuucuguuuagucucuuuuuuaagauugaacaccauugaaagcucaagaaagcugugggagaauauggcaaauuugggauuuuaauuuuuuuauuuuuguuuuuauuuuuuauuuuuccuucaacuacucuucagcugaugaugauguugcacacagaaagaaagaaagaaagaaagaaagagagaucucagaagcuaaau
......................uuccacagcuuucuugaNcu................................................................................................................................................................................................   1       1    LCK-1
......................uuccacaAcuuucuugaacu................................................................................................................................................................................................   1       1    LT-2
......................uuccacagcuuucuugaacC................................................................................................................................................................................................   3       1    JT-3
......................uuccacagcCuucuugaacu................................................................................................................................................................................................   3       1    JCK-2
......................uuccacagcuuucuugCacu................................................................................................................................................................................................   10      1    LT-1
......................uCccacagcuuucuugaacu................................................................................................................................................................................................   2       1    JCK-2
......................uuccacagcuuucuugaacG................................................................................................................................................................................................   45      1    LCK-3
......................uuccaNagcuuucuugaacu................................................................................................................................................................................................   3       1    LCK-1
......................uuccacagcuuucuugaaUu................................................................................................................................................................................................   154     1    LT-2
......................uuccacagcuuucuugaCcu................................................................................................................................................................................................   3       1    LCK-1
......................uuccacagcuuucuugGacu................................................................................................................................................................................................   8       1    LCK-3
......................uuccacagcuCucuugaacu................................................................................................................................................................................................   3       1    LT-1
......................uuUcacagcuuucuugaacu................................................................................................................................................................................................   1       1    JCK-3
......................uuccacCgcuuucuugaacu................................................................................................................................................................................................   3       1    LCK-2
......................uuccacagcuuucuugaacu................................................................................................................................................................................................   2055    0    JT-1
......................uuccacagcuuucuugaacC................................................................................................................................................................................................   39      1    LT-2
......................uuccacagcuuucuugaacu................................................................................................................................................................................................   2125    0    JCK-1
......................uuccacagcuuucuGgaacu................................................................................................................................................................................................   1       1    LT-3
......................uuccacagcuuuUuugaacu................................................................................................................................................................................................   2       1    LT-1
......................uuccacaUcuuucuugaacu................................................................................................................................................................................................   1       1    JCK-1
......................uuccacagcuuucuugaaUu................................................................................................................................................................................................   132     1    LT-1
......................uuccacagcuuucuugaaAu................................................................................................................................................................................................   2       1    LT-1
......................uuccacagcuuucuugaCcu................................................................................................................................................................................................   1       1    LT-1
......................uuccacCgcuuucuugaacu................................................................................................................................................................................................   3       1    JCK-3
......................uCccacagcuuucuugaacu................................................................................................................................................................................................   1       1    LT-1
......................uuccacagcuuucuugaacu................................................................................................................................................................................................   7087    0    LT-3
......................uuccacagcuuucCugaacu................................................................................................................................................................................................   1       1    JT-1
......................Cuccacagcuuucuugaacu................................................................................................................................................................................................   1       1    JCK-2
......................uuccCcagcuuucuugaacu................................................................................................................................................................................................   1       1    JCK-2
......................Cuccacagcuuucuugaacu................................................................................................................................................................................................   1       1    LT-2
......................uuccacagcuuucuugaaAu................................................................................................................................................................................................   6       1    LCK-3
......................uuccacCgcuuucuugaacu................................................................................................................................................................................................   3       1    JCK-1
......................uuccacagcuuucuugaaNu................................................................................................................................................................................................   1       1    LT-1
......................uuccacagcuCucuugaacu................................................................................................................................................................................................   2       1    LT-2
......................uuNcacagcuuucuugaacu................................................................................................................................................................................................   2       1    LCK-3
......................uuccacagcuuuAuugaacu................................................................................................................................................................................................   1       1    LT-2
......................uuccacaAcuuucuugaacu................................................................................................................................................................................................   1       1    LT-1
......................uuccacagcuuucuugaaAu................................................................................................................................................................................................   1       1    JCK-3
......................uuccacagcuuucuugaNcu................................................................................................................................................................................................   2       1    JCK-3
......................uuccacagcuuucuugaacu................................................................................................................................................................................................   10755   0    LCK-3
......................uuccCcagcuuucuugaacu................................................................................................................................................................................................   2       1    JT-1
......................uuccacagcuuucuugaaUu................................................................................................................................................................................................   50      1    JT-3
......................uuccacagcuuucuugaacu................................................................................................................................................................................................   2316    0    LCK-1
......................uNccacagcuuucuugaacu................................................................................................................................................................................................   2       1    JCK-3
......................uuccacaAcuuucuugaacu................................................................................................................................................................................................   1       1    LT-3
......................uuccacagcuuNcuugaacu................................................................................................................................................................................................   3       1    JCK-1
......................uuccacagcuuucuugaacu................................................................................................................................................................................................   731     0    JT-3
......................uuccacagUuuucuugaacu................................................................................................................................................................................................   1       1    JT-1
......................uuccacaAcuuucuugaacu................................................................................................................................................................................................   1       1    JT-1
......................uuccacagcuuucuugGacu................................................................................................................................................................................................   1       1    LT-3
......................uuccaAagcuuucuugaacu................................................................................................................................................................................................   1       1    JCK-3
......................uuccacagcuuucuugaCcu................................................................................................................................................................................................   3       1    JT-1
......................uuccacagcuuucuugaCcu................................................................................................................................................................................................   25      1    LT-2
......................uuccacagcuuucuugaaUu................................................................................................................................................................................................   52      1    JCK-3
......................uuccacagcuuucuugaaUu................................................................................................................................................................................................   18      1    JT-1
......................uuUcacagcuuucuugaacu................................................................................................................................................................................................   4       1    JCK-2
......................uuccacagcuuucuugaacA................................................................................................................................................................................................   137     1    LT-2
......................uuGcacagcuuucuugaacu................................................................................................................................................................................................   5       1    LT-2
......................uuccacagcuuCcuugaacu................................................................................................................................................................................................   1       1    LCK-3
......................uuccacagcuuucuugaUcu................................................................................................................................................................................................   2       1    LT-2
......................uuccacagUuuucuugaacu................................................................................................................................................................................................   1       1    LT-1
......................uuccacagcuuucuugaacA................................................................................................................................................................................................   11      1    JT-3
......................uuccacagcCuucuugaacu................................................................................................................................................................................................   1       1    JT-2
......................uuccaAagcuuucuugaacu................................................................................................................................................................................................   7       1    LCK-3
......................uuccacCgcuuucuugaacu................................................................................................................................................................................................   1       1    JT-1
......................Cuccacagcuuucuugaacu................................................................................................................................................................................................   2       1    LT-1
......................uuccacagUuuucuugaacu................................................................................................................................................................................................   1       1    JCK-2
......................uuccacagcuuucGugaacu................................................................................................................................................................................................   1       1    LCK-1
......................uuccacagcuuucuugaUcu................................................................................................................................................................................................   2       1    LT-1
......................uuccacagcuuuAuugaacu................................................................................................................................................................................................   4       1    LCK-3
```

Mature Star

```
uuugcaaguccuggucaugcuuuuccacagcuuucuugaacuuucuuucuuucuguuuagucucuuuuuuaagauugaacaccauugaaagcucaagaaagcugugggagaauauggcaaauuugggauuuuaauuuuuuuauuuuuguuuuuauuuuuuauuuuuccuucaacuacucuucagcugaugaugauguugcacacagaaagaaagaaagaaagaaagaaagagagaucucagaagcuaaau
......................uucUacagcuuucuugaacu..................................................................................................................................................................................................   3     1   LT-2
......................uuccacagcuCucuugaacu..................................................................................................................................................................................................   1     1   LCK-3
......................uuccacagcuuucuuAaacu..................................................................................................................................................................................................   1     1   LT-2
......................uuccacagcuuGcuugaacu..................................................................................................................................................................................................   1     1   JCK-1
......................uuccacagcuuucuugaacC..................................................................................................................................................................................................   2     1   JCK-2
......................uuccacagcuuucuugaacu..................................................................................................................................................................................................   6887  0   LT-2
......................uuccacagcuuucuCgaacu..................................................................................................................................................................................................   1     1   JCK-1
......................uuccacagcuuCcuugaacu..................................................................................................................................................................................................   1     1   JCK-2
......................uuccacagcuuucuugaacA..................................................................................................................................................................................................   57    1   JCK-2
......................uuccacagcuuucuugaCcu..................................................................................................................................................................................................   3     1   JCK-1
......................uuccacagcCuucuugaacu..................................................................................................................................................................................................   1     1   LT-3
......................uuccacagcuuucuugaacA..................................................................................................................................................................................................   48    1   JCK-1
......................uuccacagcuuucuugaacC..................................................................................................................................................................................................   2     1   LCK-1
......................uuccaAagcuuucuugaacu..................................................................................................................................................................................................   1     1   LCK-1
......................uuccaAagcuuucuugaacu..................................................................................................................................................................................................   3     1   LT-3
......................uuccacCgcuuucuugaacu..................................................................................................................................................................................................   6     1   JCK-2
......................uuccacagcuuuAuugaacu..................................................................................................................................................................................................   2     1   JCK-3
......................uuccacagcuuucuugaacu..................................................................................................................................................................................................   3389  0   JCK-2
......................uuccacagcuuucuGgaacu..................................................................................................................................................................................................   4     1   LCK-1
......................uuccacagcuuucGugaacu..................................................................................................................................................................................................   1     1   LCK-2
......................Cuccacagcuuucuugaacu..................................................................................................................................................................................................   4     1   LCK-3
......................uuccacagcuuuNuugaacu..................................................................................................................................................................................................   1     1   LCK-1
......................uuccacagcuuucuugCacu..................................................................................................................................................................................................   3     1   JCK-1
......................uuccacGgcuuucuugaacu..................................................................................................................................................................................................   2     1   JT-3
......................uuccacagcuuucuugaacA..................................................................................................................................................................................................   196   1   LCK-3
......................uuccCcagcuuucuugaacu..................................................................................................................................................................................................   20    1   LT-2
......................uuccacagcuuucuugaaUu..................................................................................................................................................................................................   72    1   JCK-1
......................uuccacagcuuucuugCacu..................................................................................................................................................................................................   10    1   LCK-3
......................uuccacagcuuuAuugaacu..................................................................................................................................................................................................   2     1   LT-3
......................uuccacagcuuucuugaacC..................................................................................................................................................................................................   5     1   JCK-3
......................uuccacagcuuCcuugaacu..................................................................................................................................................................................................   2     1   LT-3
......................uucUacagcuuucuugaacu..................................................................................................................................................................................................   1     1   JCK-1
......................uuccCcagcuuucuugaacu..................................................................................................................................................................................................   4     1   JCK-1
......................uuccacagcuuucuugaGcu..................................................................................................................................................................................................   2     1   LCK-3
......................uuccacagcuuCcuugaacu..................................................................................................................................................................................................   2     1   LT-2
......................uuccacagcuuucuugaacC..................................................................................................................................................................................................   7     1   JCK-1
......................uuccacagcuuucuugaacC..................................................................................................................................................................................................   3     1   JT-2
......................uuccacagcuuucuuAaacu..................................................................................................................................................................................................   3     1   LCK-3
......................uuccacagcuuucuugaacu..................................................................................................................................................................................................   544   0   JT-2
......................uuccacagcuuucuugaacu..................................................................................................................................................................................................   2309  0   JCK-3
......................uucUacagcuuucuugaacu..................................................................................................................................................................................................   3     1   LCK-3
......................uuccacagcuuucuugaaUu..................................................................................................................................................................................................   172   1   LT-3
......................uuccacagcuuucuGgaacu..................................................................................................................................................................................................   1     1   JT-1
......................uuccacagcuuucuugaacA..................................................................................................................................................................................................   87    1   LT-1
......................uuccacagcuuucuugaacG..................................................................................................................................................................................................   8     1   LT-2
......................uuccacagcuuucuugaaUu..................................................................................................................................................................................................   35    1   LCK-2
......................uuccacagcuuucuugaaAu..................................................................................................................................................................................................   2     1   LT-3
......................uuccacGgcuuucuugaacu..................................................................................................................................................................................................   1     1   LCK-3
......................uuccGcagcuuucuugaacu..................................................................................................................................................................................................   1     1   LCK-3
......................uNccacagcuuucuugaacuu.................................................................................................................................................................................................   1     1   JCK-1
......................uuccacagcuuucuugCacuu.................................................................................................................................................................................................   1     1   LCK-3
......................uuccacagcuuucuugaCcuu.................................................................................................................................................................................................   4     1   LT-2
......................uuccacagcuuucuugaacuC.................................................................................................................................................................................................   9     1   LCK-2
......................uuccacGgcuuucuugaacuu.................................................................................................................................................................................................   1     1   JCK-2
......................uuccacagcuuucuugaaUuu.................................................................................................................................................................................................   135   1   LT-3
......................uuccacGgcuuucuugaacuu.................................................................................................................................................................................................   1     1   JCK-1
......................uuccCcagcuuucuugaacuu.................................................................................................................................................................................................   5     1   LT-2
......................uCccacagcuuucuugaacuu.................................................................................................................................................................................................   1     1   LCK-3
......................uuccacagcuuucuugaaUuu.................................................................................................................................................................................................   11    1   JT-3
......................uuccacagcuuucuugaacAu.................................................................................................................................................................................................   6     1   LT-2
......................uucNacagcuuucuugaacuu.................................................................................................................................................................................................   1     1   JCK-3
......................uuccacGgcuuucuugaacuu.................................................................................................................................................................................................   7     1   JT-2
......................uuccacagcuuucuugaaUuu.................................................................................................................................................................................................   112   1   LCK-3
......................uuccacagcuuucuugaacNu.................................................................................................................................................................................................   2     1   JCK-1
......................uuccacagcuuucuugaaUuu.................................................................................................................................................................................................   86    1   LT-1
......................uuccacagcuuCcuugaacuu.................................................................................................................................................................................................   1     1   LT-2
......................uuccacagcuuucuugaacuA.................................................................................................................................................................................................   75    1   JCK-1
......................uCccacagcuuucuugaacuu.................................................................................................................................................................................................   1     1   LT-2
......................uuccacagcuuucuugCacuu.................................................................................................................................................................................................   2     1   LT-3
......................uuccacGgcuuucuugaacuu.................................................................................................................................................................................................   11    1   JT-3
```

```
                      Mature                                                                     Star
uuugcaaguccuggucaugcuuuuccacagcuuucuugaacuuucuuucuuucuguuuagucucuuuuuuaagauugaacaccauugaaagcucaagaaagcugugggagaauauggcaaauuugggauuuuaauuuuuuuauuuuuguuuuuauuuuuuauuuuuccuucaacuacucuucagcugaugaugauguugcacacagaaagaaagaaagaaagaaagaaagagagaucucagaagcuaaau
......................uuccacagcuuucuugaacuu...............................................................................................................................................................................   2955   0   JCK-3
......................uuccacagcuuucuugaacuu...............................................................................................................................................................................   191    0   LCK-2
......................uuccacagcuuucuugaacuC...............................................................................................................................................................................   53     1   JCK-1
......................uucGacagcuuucuugaacuu...............................................................................................................................................................................   1      1   JCK-1
......................uuccacagcuuucuugaacuu...............................................................................................................................................................................   1431   0   LT-2
......................uuccacagcuuucuugaacAu...............................................................................................................................................................................   2      1   JCK-1
......................uuccacCgcuuucuugaacuu...............................................................................................................................................................................   3      1   JCK-3
......................uuccacagAuuucuugaacuu...............................................................................................................................................................................   1      1   JCK-1
......................uuccacagcuuucuugaacAu...............................................................................................................................................................................   1      1   LT-1
......................uuccacagcuuucuugCacuu...............................................................................................................................................................................   1      1   JT-1
......................uuccacagcuuucuugaacGu...............................................................................................................................................................................   1      1   JCK-2
......................uuccaAagcuuucuugaacuu...............................................................................................................................................................................   3      1   JCK-3
......................uuccacagcuuucuuAaacuu...............................................................................................................................................................................   1      1   LT-1
......................uuccaUagcuuucuugaacuu...............................................................................................................................................................................   1      1   JCK-1
......................uuccacagcuuucuugaacuN...............................................................................................................................................................................   4      1   JCK-1
......................uuccacagcuuucuugaGcuu...............................................................................................................................................................................   4      1   JCK-1
......................uuccacagcuuucuuAaacuu...............................................................................................................................................................................   2      1   LCK-3
......................uuccacagcuuucuuAaacuu...............................................................................................................................................................................   1      1   JCK-1
......................uuUcacagcuuucuugaacuu...............................................................................................................................................................................   6      1   JCK-2
......................uuccacagcuuucuugaacuu...............................................................................................................................................................................   157    0   LCK-1
......................uuccacagcuuucuugaacuC...............................................................................................................................................................................   33     1   LT-1
......................uuccacagcuuucuugaacuC...............................................................................................................................................................................   18     1   JT-1
......................uuccacagcuuucuugaaUuu...............................................................................................................................................................................   88     1   JCK-2
......................uuccacagcuuucCugaacuu...............................................................................................................................................................................   1      1   LT-3
......................uuccacagcuuucuugaacuA...............................................................................................................................................................................   97     1   JT-1
......................uuccacagcCuucuugaacuu...............................................................................................................................................................................   1      1   JCK-3
......................uuccacagcuuucuugaacuu...............................................................................................................................................................................   97     0   JT-2
......................uuccCcagcuuucuugaacuu...............................................................................................................................................................................   2      1   JCK-3
......................uuccacagcCuucuugaacuu...............................................................................................................................................................................   2      1   LT-1
......................uuccacCgcuuucuugaacuu...............................................................................................................................................................................   3      1   JCK-2
......................uuccacagcuuucuugaacuu...............................................................................................................................................................................   2349   0   JCK-1
......................uuccacagcuuucuugaCcuu...............................................................................................................................................................................   5      1   JCK-2
......................uNccacagcuuucuugaacuu...............................................................................................................................................................................   4      1   JCK-2
......................uuccacagcuuuAuugaacuu...............................................................................................................................................................................   1      1   LT-2
......................uuccacagcuuucuugaacuA...............................................................................................................................................................................   240    1   LCK-3
......................uuccacagcuuucuCgaacuu...............................................................................................................................................................................   5      1   LT-2
......................uuccCcagcuuucuugaacuu...............................................................................................................................................................................   1      1   LCK-3
......................uuccacagcuuucuugaCcuu...............................................................................................................................................................................   1      1   LT-3
......................uuccacagcuuucuugaacuC...............................................................................................................................................................................   64     1   JCK-2
......................uucUacagcuuucuugaacuu...............................................................................................................................................................................   4      1   JCK-2
......................uuccacagcuuucuugaacuu...............................................................................................................................................................................   1769   0   LT-3
......................uuccacagcuuucuugaacAu...............................................................................................................................................................................   6      1   LT-3
......................uuccacagcuuucuugaaUuu...............................................................................................................................................................................   48     1   LCK-2
......................uuccacagcuuucuugaaAuu...............................................................................................................................................................................   1      1   LT-1
......................uuccacagcuuucuugaacAu...............................................................................................................................................................................   8      1   LCK-3
......................uuccaNagcuuucuugaacuu...............................................................................................................................................................................   2      1   LCK-1
......................uuccacagcuuucuugaacuA...............................................................................................................................................................................   20     1   LCK-1
......................uuccacagcuuucuugaCcuu...............................................................................................................................................................................   3      1   LCK-3
......................uuccacagcuuucuugaacuA...............................................................................................................................................................................   67     1   JCK-3
......................uuccacagcuuucuugCacuu...............................................................................................................................................................................   1      1   LT-1
......................uuccacagcuuucuugaacuu...............................................................................................................................................................................   2427   0   LCK-3
......................uuccacagNuuucuugaacuu...............................................................................................................................................................................   1      1   JCK-3
......................uuccCcagcuuucuugaacuu...............................................................................................................................................................................   6      1   JCK-2
......................uuccacagcuuucuugaacuC...............................................................................................................................................................................   68     1   JCK-3
......................uuccacagcuuuUuugaacuu...............................................................................................................................................................................   1      1   LT-1
......................uuccacagcuuucuugaaUuu...............................................................................................................................................................................   61     1   JCK-3
......................uuccacagcuuucuugaaUuu...............................................................................................................................................................................   29     1   LCK-1
......................uuccacagcuuucuugaaUuu...............................................................................................................................................................................   50     1   JCK-1
......................uuccacagcuuucuugaacuA...............................................................................................................................................................................   125    1   JCK-2
......................uuccacagcuuucuugaaAuu...............................................................................................................................................................................   5      1   LT-2
......................uuccacagcuuucuuAaacuu...............................................................................................................................................................................   1      1   LCK-2
......................uuccacCgcuuucuugaacuu...............................................................................................................................................................................   3      1   LCK-3
......................Cuccacagcuuucuugaacuu...............................................................................................................................................................................   1      1   LT-3
......................uuccacagcuuucuugaacAu...............................................................................................................................................................................   3      1   JCK-3
......................uuccacagcuuucuugaacuA...............................................................................................................................................................................   2      1   JT-2
......................uuccacagcuuucCugaacuu...............................................................................................................................................................................   1      1   JCK-2
......................uuccacagcuuuUuugaacuu...............................................................................................................................................................................   1      1   JCK-2
......................uuccacagcuuucCugaacuu...............................................................................................................................................................................   1      1   JCK-1
......................uuccaAagcuuucuugaacuu...............................................................................................................................................................................   1      1   LT-2
......................uuccacagcuuucuugaacuA...............................................................................................................................................................................   148    1   LT-3
```

Mature Star

uuugcaaguccuggucaugcuuuuccacagcuuucuugaacuuucuuucuuucuguuuagucucuuuuuuaagauugaacaccauugaaagcucaagaaagcugugggagaauauggcaaauuugggauuuuaauuuuuuuauuuuuguuuuuauuuuuuauuuuuccuucaacuacucuucagcugaugaugauguugcacacagaaagaaagaaagaaagaaagaaagagagaucucagaagcuaaau

......................uuccCcagcuuucuugaacuu...	2	1	JCK-1
......................uuUcacagcuuucuugaacuu...	2	1	JT-2
......................uuccacagcuuucuugaacuC...	44	1	LT-2
......................uuUcacagcuuucuugaacuu...	2	1	JCK-3
......................uuccacagcuuucuugaacuu...	145	0	JT-3
......................uuccacagcuuucuugaaUuu...	3	1	JT-2
......................uuccacagcuuucuugaaAuu...	3	1	LCK-3
......................uuccacagcuuucuugaCcuu...	2	1	JCK-3
......................uuccacagcuuucuugaacGu...	1	1	LT-1
......................uNccacagcuuucuugaacuu...	1	1	JT-1
......................uNccacagcuuucuugaacuu...	6	1	JCK-3
......................uCccacagcuuucuugaacuu...	1	1	LCK-2
......................uuccacagcuCucuugaacuu...	1	1	JT-2
......................uCccacagcuuucuugaacuu...	1	1	JCK-2
......................uuccacagcuuuAuugaacuu...	1	1	LCK-3
......................uuccacaAcuuucuugaacuu...	1	1	LCK-3
......................uuccacCgcuuucuugaacuu...	3	1	LT-3
......................uucNacagcuuucuugaacuu...	2	1	JCK-1
......................uuccacagcuuucuugaacuC...	53	1	LCK-3
......................uuccacagcuuucuugaacuC...	54	1	LT-3
......................uuccacaAcuuucuugaacuu...	1	1	LT-1
......................uuccaUagcuuucuugaacuu...	3	1	JCK-3
......................uuccaAagcuuucuugaacuu...	5	1	JCK-2
......................uuccCcagcuuucuugaacuu...	3	1	LT-3
......................uNccacagcuuucuugaacuu...	1	1	LT-2
......................uuccacagcuuucuugaacuu...	954	0	LT-1
......................uuccacagcuuucuugaCcuu...	2	1	JT-1
......................uuccacagcuuucuugaacuA...	66	1	LT-1
......................uuccacCgcuuucuugaacuu...	1	1	LCK-2
......................uuccacaAcuuucuugaacuu...	1	1	JCK-3
......................uuccacagcuuucuugaacAu...	5	1	JCK-2
......................uuccacagcuuucuugCacuu...	2	1	JCK-3
......................uuccacagcuuucuugaacuu...	502	0	JT-1
......................uuUcacagcuuucuugaacuu...	1	1	JT-3
......................uuccacagcuuNcuugaacuu...	3	1	JCK-1
......................uuccacagcuuucuugaacuu...	3888	0	JCK-2
......................uuccaUagcuuucuugaacuu...	1	1	LT-3
......................uuccacaAcuuucuugaacuu...	1	1	LT-2
......................uuccacagcuuucuugaacGu...	1	1	LT-3
......................uuccacagcuuucuugCacuu...	5	1	JCK-2
......................uuccacCgcuuucuugaacuu...	1	1	JT-1
......................uuccacagcuuucuugaCcuu...	3	1	JCK-1
......................uuccacagcuuuAuugaacuu...	1	1	LT-3
......................uuccacagcuuucuugaacuA...	5	1	JT-3
......................uuUcacagcuuucuugaacuu...	1	1	JCK-1
......................uuccacagcuuucuugCacuu...	2	1	JCK-1
......................uuccaAagcuuucuugaacuu...	2	1	JCK-1
......................uuccacCgcuuucuugaacuu...	5	1	JCK-1
......................uuNcacagcuuucuugaacuu...	4	1	LT-2
......................uucUacagcuuucuugaacuu...	1	1	JCK-3
......................uuccacagcuuucuugaaUuu...	101	1	LT-2
......................Cuccacagcuuucuugaacuu...	3	1	JCK-3
......................uuccacagcuuucuugaacuC...	6	1	LCK-1
......................uucNacagcuuucuugaacuu...	1	1	JT-1
......................uuccacagcuuucuugCacuu...	2	1	LT-2
......................uuccacagcuuucuugaacuA...	134	1	LT-2
......................uuccacagcuuucuugaacuA...	23	1	LCK-2
......................uuccacagcuuuAuugaacuu...	3	1	JCK-3
......................uuccacagcuuucuugaacCu...	1	1	LT-2
......................uuccaUagcuuucuugaacuuu...	1	1	LT-1
......................uuccacagcuuucuugaacuuu...	64	0	JT-3
......................uuccacagcuuucuugaacCuu...	1	1	JCK-1
......................uuccacCgcuuucuugaacuuu...	2	1	LT-3
......................uuccacagcuuucuugaacuuu...	250	0	LCK-1
......................uuccacagcuuucuugaacuuC...	65	1	JCK-1
......................uuccacagcuuucuugaacuuu...	1432	0	LT-2
......................uuccacagcuuucuugaacuuC...	114	1	LT-2
......................uuccacagcuuucuugaacuuA...	77	1	JCK-3
......................uuccacagcuuucuugaacuuA...	50	1	JCK-1
......................uuccacagcuuucuugaaUuuu...	13	1	LCK-2

Mature / Star			
uuugcaaguccuggucaugcuuuuccacagcuuucuugaacuuucuuucuuucuguuuagucucuuuuuuaagauugaacaccauugaaagcucaagaaagcuguggagaauauggcaaauuugggauuuuaauuuuuuuauuuuuguuuuuauuuuuuauuuuuccuucaacuacucuucagcugaugaugauguugcacacagaaagaaagaaagaaagaaagaaagagagaucucagaagcuaaau			
......................uuccacagcCuucuugaacuuu...	4	1	JCK-3
......................uuccacagcuuucuugaacuuN...	1	1	LT-2
......................uuccacagcuuucuugaacuuA...	128	1	JCK-2
......................uuccacagcuGucuugaacuuu...	2	1	JCK-1
......................uuccacagcuuucuugaacuuA...	31	1	LCK-2
......................uuccacagcuuucuugaacuAu...	11	1	LT-1
......................uuccacagcuCucuugaacuuu...	1	1	LT-1
......................uuccCcagcuuucuugaacuuu...	4	1	LT-3
......................uCccacagcuuucuugaacuuu...	3	1	LT-3
......................uuccCcagcuuucuugaacuuu...	1	1	JCK-1
......................uuccaAagcuuucuugaacuuu...	1	1	JCK-1
......................uuccacCgcuuucuugaacuuu...	3	1	JCK-3
......................uuccacagcuuucuugaacAuu...	16	1	LCK-3
......................uNccacagcuuucuugaacuuu...	1	1	JCK-1
......................uuccacagcuuNcuugaacuuu...	3	1	JCK-1
......................Cuccacagcuuucuugaacuuu...	1	1	JCK-3
......................uuccacagcuuucuugaacCuu...	3	1	JCK-2
......................uuccaNagcuuucuugaacuuu...	1	1	LCK-1
......................uuccacagcuuucuCgaacuuu...	3	1	LT-3
......................uuccacCgcuuucuugaacuuu...	2	1	LT-1
......................uuccCcagcuuucuugaacuuu...	1	1	JCK-3
......................uuccacagcuuucuugaacAuu...	4	1	JT-3
......................uuccacagcuuucuugaaUuuu...	7	1	JT-1
......................uuccacagcuuucuugaacAuu...	26	1	LT-2
......................uuccacagcuuucuugaacuuu...	377	0	JT-1
......................uuccacagcuuucuugaacuuA...	49	1	JT-1
......................uuccacagcuuucNugaacuuu...	1	1	LCK-1
......................uuccacagcuuucuugaacuuA...	17	1	JT-3
......................uuccacagcuGucuugaacuuu...	1	1	LT-2
......................uuccacagcuuucuugaaAuuu...	2	1	LT-3
......................uuccacagcuuucCugaacuuu...	1	1	JCK-2
......................uuccacagcuuucuugaCcuuu...	3	1	LT-2
......................uNccacagcuuucuugaacuuu...	2	1	LCK-3
......................uuccacagcuuucuugaacuuC...	73	1	LT-1
......................uuccacagcCuucuugaacuuu...	2	1	JCK-1
......................uuccacagcuuucuugaacCuu...	1	1	LT-3
......................uuccacagcuuucuugaacuCu...	5	1	LCK-3
......................uuccacagcuuucuugaacuuG...	3	1	LT-3
......................uuccacagcuuucuugCacuuu...	2	1	LT-2
......................uuccacagcuuucuugaCcuuu...	2	1	JCK-2
......................uuccacagcuuucuugaacuuu...	70	0	JT-2
......................uuccacagcuuucuugaaAuuu...	3	1	JCK-2
......................uuccCcagcuuucuugaacuuu...	3	1	LT-2
......................uuccacagcuuucuugaaUuuu...	36	1	JCK-3
......................uuccacagcuuucuugaacuuC...	118	1	LT-3
......................uuccacagcuuucuugaacuNu...	1	1	JCK-1
......................uuccacCgcuuucuugaacuuu...	1	1	JT-1
......................uuccacagcuuucuugaacuAu...	6	1	JCK-3
......................uuccacagcuuucuugaacuAu...	12	1	LT-3
......................uuccacagcCuucuugaacuuu...	2	1	JCK-2
......................uuccacagcuuucuugaacuCu...	1	1	LT-1
......................uuccacagcuuucuugaaUuuu...	27	1	JCK-1
......................uuccacagcuuucuugaacuuN...	1	1	JCK-1
......................uuccacagcuuucuugaCcuuu...	1	1	LCK-3
......................uuccacagcuuucuugaacuuG...	2	1	JCK-2
......................uuccacagcuuuAuugaacuuu...	2	1	JCK-1
......................uuccacagcuuucuugaacuAu...	17	1	LCK-3
......................uuccacagcuuucuugaacuCu...	3	1	LT-3
......................uuccacagcuuucuugaacAuu...	12	1	LT-1
......................uucUacagcuuucuugaacuuu...	1	1	LCK-3
......................uuccacagcuuucuugaacuuC...	8	1	JT-2
......................uuccacagcuuucuugaacGuu...	3	1	JCK-1
......................uuccacagcuuucuugCacuuu...	1	1	LCK-2
......................uuccacagcuuucuugaacuuC...	55	1	JCK-3
......................uuccacagcuuucuugaaAuuu...	2	1	JT-3
......................Cuccacagcuuucuugaacuuu...	1	1	LT-3
......................uuccacagcuuucuugaCcuuu...	2	1	LT-1
......................uuccacagcuuucuugCacuuu...	2	1	LT-1
......................uuccacagcuuucuugaaUuuu...	1	1	JT-3
......................uuccacagcuuucuugaacuuu...	1108	0	JCK-3

Mature **Star**

uuugcaaguccuggucaugcuu**uuccacagcuuucuugaacu**uucuuucuuucuguuuagucucuuuuuuaagauugaacaccauugaaag**cucaagaaagcugugggaga**auauggcaaauuugggauuuuaauuuuuuuauuuuuguuuuuauuuuuuauuuuuccuucaacuacucuucagcugaugaugauguugcacacagaaagaaagaaagaaagaaagaaagagagaucucagaagcuaaau

......................uuccacagcuuucuugaacAuu	6	1	JT-2
......................uNccacagcuuucuugaacuuu	1	1	JCK-3
......................uucNacagcuuucuugaacuuu	1	1	JCK-1
......................uuccCcagcuuucuugaacuuu	4	1	LCK-3
......................uuNcacagcuuucuugaacuuu	4	1	LT-2
......................uuccacagcuuucuugaaAuuu	2	1	JCK-3
......................uuccacagcuuucuugaacuuu	2105	0	LCK-3
......................uuccacagcuuucuugCacuuu	1	1	JCK-3
......................uuccCcagcuuucuugaacuuu	2	1	JCK-2
......................uuccacagcuuucuugaaUuuu	63	1	JCK-2
......................uuccacagcuuucuugaacuuC	253	1	LCK-3
......................uuccacagcuuucuugaacuCu	5	1	JCK-3
......................uuccacagcuuucuugaacCuu	1	1	LCK-1
......................uuccacagcuuucuugaacuuA	121	1	LT-2
......................uuccaAagcuuucuugaacuuu	1	1	JCK-3
......................uuccacagcuuucuugaacGuu	1	1	LT-3
......................uuccacagcuuucuugaacuuC	41	1	JT-1
......................uuccGcagcuuucuugaacuuu	1	1	LT-2
......................uuccacagcuuucuugaacuuA	157	1	LCK-3
......................uuccacagcuuucuuUaacuuu	1	1	LT-3
......................uuccacCgcuuucuugaacuuu	1	1	LCK-3
......................uuccacagcuuucuugaacGuu	8	1	JCK-2
......................uuccacagcCuucuugaacuuu	2	1	LT-2
......................uuccacagcuuucuugaCcuuu	1	1	LCK-2
......................uuccacagcuuucuugCacuuu	1	1	LCK-3
......................uuccacagcuuucuugaacuuu	1512	0	LT-3
......................uGccacagcuuucuugaacuuu	1	1	LCK-3
......................uuccacaAcuuucuugaacuuu	2	1	LT-2
......................uuGcacagcuuucuugaacuuu	2	1	LT-2
......................uuccacagcuuucuugaacuAu	16	1	JCK-2
......................uuccacagcuuucuugaaUuuu	41	1	LT-1
......................Cuccacagcuuucuugaacuuu	1	1	JCK-1
......................uNccacagcuuucuugaacuuu	1	1	JCK-2
......................Cuccacagcuuucuugaacuuu	1	1	JT-1
......................uuccacagcuuucuugCacuuu	1	1	JT-3
......................uuccacagcuuucuugaacuCu	2	1	JCK-1
......................uuccacagcuuucuugCacuuu	1	1	JCK-1
......................uuccacaAcuuucuugaacuuu	1	1	JT-3
......................uuccacagcuuucuugaacuuA	62	1	LT-1
......................uuccacagcuuucuugaaUuuu	3	1	JT-2
......................uuUcacagcuuucuugaacuuu	1	1	JCK-2
......................uuccacagcuuucuugaacAuu	12	1	JCK-3
......................uuccacagcuuucuugaacuuG	7	1	LT-2
......................uuccacagcuuucuugaaUuuu	32	1	LT-3
......................uuccacagcuuucuugaaUuuu	39	1	LCK-3
......................uuccacagcuuucuugaacuuG	2	1	LT-1
......................uuccacagcuuucuugCacuuu	1	1	JCK-2
......................uuccacagcuuucuugaacuuC	89	1	JCK-2
......................uuUcacagcuuucuugaacuuu	1	1	LT-3
......................uuccacagcuuucuugaacuuA	21	1	LCK-1
......................uuccacagcuuGcuugaacuuu	2	1	JCK-1
......................uuccaAagcuuucuugaacuuu	1	1	JCK-2
......................uuUcacagcuuucuugaacuuu	7	1	LT-2
......................uuccacagcuuNcuugaacuuu	1	1	JT-1
......................uuccacCgcuuucuugaacuuu	2	1	JCK-2
......................uuccacagcuuucuugaacuuC	12	1	JT-3
......................uuccacagcuuCcuugaacuuu	1	1	LT-2
......................uuccacagcuuucuugaacAuu	14	1	JCK-1
......................uuccacCgcuuucuugaacuuu	1	1	JCK-1
......................uuccCcagcuuucuugaacuuu	1	1	LT-1
......................uuccacagcuuuAuugaacuuu	4	1	LT-2
......................uuccacagcuuucuugaacuAu	13	1	LT-2
......................uuccacagcuuucuugaacuuu	899	0	JCK-1
......................uuccacagcuuucuugaacuuA	121	1	LT-3
......................uuccacagcuuucuugaCcuuu	2	1	LT-3
......................uuccacCgcuuucuugaacuuu	2	1	LCK-2
......................uuccacagcuuucuugaaUuuu	22	1	LCK-1
......................uuccacagcuuucuugaacCuu	3	1	JCK-3
......................uuUcacagcuuucuugaacuuu	1	1	JCK-1
......................uuccacagcuuucuugaacAuu	36	1	JCK-2

Mature **Star**

uuugcaaguccuggucaugcuuuuccacagcuuucuugaacuuucuuucuuucuguuuagucucuuuuuuaagauugaacaccauugaaagcucaagaaagcuguggagaauauggcaaauuugggauuuuaauuuuuuuauuuuuguuuuuauuuuuuauuuuuccuucaacuacucuucagcugaugaugauguugcacacagaaagaaagaaagaaagaaagaaagagagaucucagaagcuaaau

uuccacagcuuucuugaacuuu	246	0	LCK-2
uuccacagcuuucuugaacuCu	1	1	LT-2
uuccacagcuuucuugaaAuuu	4	1	JCK-1
uuccacCgcuuucuugaacuuu	8	1	LT-2
uCccacagcuuucuugaacuuu	1	1	LT-1
uuccacagcuuucuugaaUuuu	37	1	LT-2
uuccacagcuuucuugaacuAu	3	1	JCK-1
uuccacagcuuucuugaacAuu	29	1	LT-3
uCccacagcuuucuugaacuuu	1	1	JCK-3
uuccacagcuuucuugaacuuA	10	1	JT-2
uuccacagcuuucuugaacuCu	6	1	JCK-2
uuccacagcuuucuugaacuuu	1496	0	JCK-2
uuccacagcuuuAuugaacuuu	1	1	LT-1
uuccacagcuuucuugaacuNu	1	1	JT-2
uuccacagcuuucuugaacuuu	1068	0	LT-1
uuccacagcuuucuugaacuuuA	11	1	JT-2
uuccacagcuuucuugaacuuuG	9	1	LT-2
uuccacagcuuucuugaacuuuA	166	1	LT-3
uuccacagcuuucuugaacuuuG	11	1	LT-3
uuccacagcuuucuugaacuAuc	1	1	LT-3
uuccacGgcuuucuugaacuuuc	1	1	LT-2
uuccacagcuuucuugaacuuuA	119	1	LT-1
uuccacagcuuucuugaacuuuG	4	1	JCK-3
uuccacagcuuucuugaacuuuU	134	1	LCK-2
uuccacagcuuucuugaacAuuc	3	1	LT-3
uuccacagcuuucuugaaUuuuc	6	1	LCK-3
uuccacagcuuucuugaacuuAc	1	1	JCK-3
uuccacagcuuucuugaacuuuc	50	0	JCK-2
uuccacagcuuucuugaacuuAc	1	1	JT-2
uuccacagcuuucuugaacuuuA	301	1	LCK-3
uuccacagcuuucuugaacuuuU	324	1	LT-3
uuccacagcuuucuugaacuuuG	4	1	JCK-1
uuccacagcuuucuugaacuuuU	490	1	LCK-3
uuccacagcuuucuugaacuuuc	47	0	LT-3
uuccacagcuuucuugaacuuuU	369	1	JCK-3
uuccacagcuuucuugaacAuuc	2	1	LT-2
uuccacagcuuucuugaacuuuA	48	1	LCK-2
uuccacagcuuucuugaaUuuuc	15	1	LCK-1
uuccacagcuuucuugaacuuuU	18	1	JT-3
uuccacagcuuucuugaaUuuuc	13	1	LCK-2
uuccacagcuuucuugaacAuuc	1	1	JCK-3
uuccacagcuuucuugaacuuuA	155	1	JCK-3
uuNcacagcuuucuugaacuuuc	1	1	LT-2
uuccacCgcuuucuugaacuuuc	1	1	LCK-3
uuccacagcuuucuugaaUuuuc	4	1	LT-1
uuccacagcuuucuugaaAuuuc	1	1	LT-3
uuccacagcuuucuugaacuuuG	8	1	JCK-2
uuccacagcuuucuugaacuuuc	28	0	LT-1
uuccacagcuuucuugaacuuuc	3	0	JT-2
uuccacagcuuucuugaacuuuU	492	1	JCK-2
uuccacagcuuucuugaacuuuA	47	1	JT-1
uuccacagcuuucuugaacuuuG	9	1	LCK-3
uuccacagcuuucuugaacAuuc	10	1	LCK-3
uuccacagcuuucuugaacuuuc	33	0	JCK-1
uuccacCgcuuucuugaacuuuc	1	1	LT-2
uuccacagcuuucuugaacuuuc	61	0	LT-2
uuccacagcuuucuugaacuuuU	294	1	JCK-1
uuccacagcuuucuugaacuAuc	4	1	LT-1
uuccacagcuuucuugaacuuuU	376	1	LT-2
uuccacagcuuucuugaacuuuU	101	1	JT-1
uuccacagcuuucuugaaUuuuc	5	1	JCK-3
uuccacagcuuucuugaacuuuc	16	0	JT-1
uuccacagcuuucuugaacuuuA	16	1	JT-3
uuccacagcuuucuugaacuuAc	5	1	JT-3
uuccacagcuuucuugaacuuuA	144	1	JCK-1
uuccacagcuuucuugaacuuAc	1	1	LT-2
uuccacagcuuucuugCacuuuc	1	1	LT-2
uuccacagcuuucuugaacuuuU	306	1	LT-1
uuccacagcuuucuugaaUuuuc	3	1	JCK-2
uuccacagcuuucuugaacuuCc	1	1	LT-1

Mature / Star			
uuugcaaguccuggucaugcuu**uuccacagcuuucuugaacu**uucuuucuuucuguuuagucucuuuuuuaagauugaacaccauugaaag**cucaagaaagcugugggaga**auauggcaaauuugggauuuuaauuuuuuuauuuuuguuuuuauuuuuuauuuuuccuucaacuacucuucagcugaugaugauguugcacacagaaagaaagaaagaaagaaagaaagagagaucucagaagcuaaau			
......................uuccacagcuuucuugaacuuuU.......	9	1	JT-2
......................uuccacagcuuucuugaacuuuc.......	40	0	JCK-3
......................uuccacagcuuucuugaacuuuA.......	177	1	JCK-2
......................uuccacagcuuucuugaacuuAc.......	1	1	LT-3
......................uuccacagcuuucuugaacuAuc.......	7	1	LT-2
......................uuccacagcuuucuugaacuuuA.......	40	1	LCK-1
......................uuccacagcuuucuugCacuuuc.......	1	1	LCK-3
......................uuccacagcuuucuugaacAuuc.......	4	1	LT-1
......................uuccacagcuuucuugaacuuuU.......	140	1	LCK-1
......................uuccacagcuuucuugaacuuuc.......	97	0	LCK-3
......................uuccCcagcuuucuugaacuuuc.......	1	1	LT-2
......................uuccacagcuuucuugaacuuuc.......	3	0	JT-3
......................uuccacagcuuucuugaacuuuA.......	230	1	LT-2
......................uuccacagcuuucuugaacuuuG.......	13	1	LT-1
......................uuccacagcuuucuugaacAuuc.......	1	1	JCK-1
......................uuccacagcuuucuugaacuuuAu......	2	1	LT-2
......................uuccacagcuuucuugaacuuAcu......	1	1	LT-3
......................uuccacagcuuucuugaacuuuUu......	89	1	JCK-2
......................uuccacagcuuucuugaacuuucA......	4	1	JCK-3
......................uuccacagcuuucuugaacuuAcu......	2	1	JCK-2
......................uuccacagcuuucuugaacuuuUu......	13	1	LCK-3
......................uuccacagcuuucuugaacuuuAu......	4	1	JT-1
......................uuccacagcuuucuugaacuuuUu......	49	1	JCK-3
......................uuccacagcuuucuugaacuuuAu......	7	1	JCK-3
......................uuccacagcuuucuugaacuuuUu......	15	1	LT-2
......................uuccacagcuuucuugaacuuuAu......	6	1	JCK-2
......................uuccacagcuuucuugaacuuuAu......	2	1	LT-1
......................uuccacagcuuucuugaacuuuUu......	13	1	LT-3
......................uuccacagcuuucuugaacuuucA......	2	1	JCK-2
......................uuccacagcuuucuugaacuuuUu......	19	1	LT-1
......................uuccacagcuuucuugaacuuuAu......	2	1	JCK-1
......................uuccacagcuuucuugaacuuuAu......	6	1	LT-3
......................uuccacagcuuucuugaacuuuUu......	47	1	JCK-1
......................uuccacagcuuucuugaacuAucu......	1	1	JCK-3
......................uuccacagcuuucuugaacuuuAu......	5	1	LCK-3
......................uuccacagcuuucuugaacuuucA......	1	1	LT-1
......................uuccacagcuuucuugaacuuucA......	1	1	JCK-1
......................uuccacagcuuucuugaacuAucu......	1	1	JCK-1
......................uuccacagcuuucuugaacuuAcu......	1	1	LT-1
......................uuccacagcuuucuugaacuuucA......	6	1	JT-1
......................uuccacagcuuucuugaacuuucu......	2	0	LCK-3
......................uuccacagcuuucuugaacuuucu......	3	0	LT-1
......................uuccacagcuuucuugaacuuAcu......	2	1	LT-2
......................uuccacagcuuucuugaacuuCcu......	1	1	LCK-3
......................uuccacagcuuucuugaacuuucA......	1	1	LT-3
......................uuccacagcuuucuugaacuAucu......	4	1	JCK-2
......................uuccacagcuuucuugaacuAucu......	5	1	LCK-3
......................uuccacagcuuucuugaacuuucA......	6	1	LT-2
......................uuccacagcuuucuugaacuuuUuu.....	2	1	LT-3
......................uuccacagcuuucuugaacuuuAuu.....	12	1	JCK-3
......................uuccacagcuuucuugaacuuuUuu.....	2	1	LCK-3
......................uuccacagcuuucuugaacuuuAuu.....	7	1	LCK-3
......................uuccacagcuuucuugaacuuuUuu.....	9	1	JCK-1
......................uuccacagcuuucuugaacuuucuA.....	1	1	LT-2
......................uuccacagcuuucuugaacuuuAuu.....	7	1	JCK-1
......................uuccacagcuuucuugaacuuuUuu.....	1	1	LT-1
......................uuccacagcuuucuugaacuuuAuu.....	3	1	LT-3
......................uuccacagcuuucuugaacuuucuC.....	1	1	LT-3
......................uuccacagcuuucuugaacuuuUuu.....	6	1	JCK-3
......................uuccacagcuuucuugaacuuAcuu.....	3	1	JCK-2
......................uuccacagcuuucuugaacuuuUuu.....	12	1	JCK-2
......................uuccacagcuuucuugaacuuuAuu.....	4	1	LT-2
......................uuccacagcuuucuugaacuuuGuu.....	1	1	LT-3
......................uuccacagcuuucuugaacuuuUuu.....	5	1	LT-2
......................uuccacagcuuucuugaacuuuAuu.....	5	1	LT-1
......................uuccacagcuuucuugaacuuuAuu.....	8	1	JCK-2
......................uuccacagcuuucuugaacuuuAuuu....	3	1	LCK-3
......................uuccacagcuuucuugaacuuuUuuu....	2	1	LCK-3
......................uuccacagcuuucuugaacuuuAuuu....	5	1	JCK-3
......................uuccacagcuuucuugaacuuuAuuu....	3	1	JCK-1

Mature **Star**

uuugcaaguccuggucaugcuuuuccacagcuuucuugaacuuucuuucuuucuguuuagucucuuuuuuaagauugaacaccauugaaagcucaagaaagcugugggagaauauggcaaauuugggauuuuaauuuuuuuauuuuuguuuuuauuuuuuauuuuuccuucaacuacucuucagcugaugaugauguugcacacagaaagaaagaaagaaagaaagaaagagagaucucagaagcuaaau			
......................uuccacagcuuucuugaacuuuAuuu	3	1	JCK-2
......................uuccacagcuuucuugaacuuucuuu	1	0	JCK-1
.......................uccacagcuuucuugaac	6	0	JT-1
.......................uccacagcuuucuugaac	2	0	JCK-3
.......................uccacagcuuucuugaac	11	0	LT-3
.......................uccacagcuuucuugaac	2	0	JCK-2
.......................uccacaUcuuucuugaac	1	1	LT-3
.......................uccacagcuuucuugaac	13	0	LCK-3
.......................Cccacagcuuucuugaac	1	1	LT-1
.......................uccacagcuuucuugaac	2	0	JT-2
.......................uccacagcuuucuugaac	10	0	LT-2
.......................uccacagcuuucuugaac	4	0	JT-3
.......................uccacagcuuucuugaac	4	0	LT-1
.......................ucNacagcuuucuugaac	1	1	LT-2
.......................uccacagcuCucuugaac	1	1	LT-3
.......................uccacagcuuucuugaacu	7	0	JCK-3
.......................uccacagcuuucuugaacu	12	0	LCK-3
.......................uccacagcuuucuugaacu	18	0	LT-3
.......................uccacagcuuucuugaacu	2	0	JCK-1
.......................uccacagcuuucuugaacu	7	0	LT-1
.......................uccacagcuuucuugaacA	1	1	LT-3
.......................uccacagcuuucuugaaAu	3	1	LT-2
.......................uccacagcuuucuugaacu	10	0	JCK-2
.......................uccacagcuuucuugaacu	29	0	LCK-2
.......................uccacagcuuucuugaaUu	1	1	LT-1
.......................uccacagcuuucuugaacA	1	1	LT-1
.......................uccacagcuuucuugaacu	2	0	LT-2
.......................uccacagcuuucuugaacu	53	0	LCK-1
.......................uccacagcuuucuugaaUuu	1	1	LT-3
.......................uccacagcuuucuugaacuu	5	0	JCK-3
.......................uccacagcuuucuugaacuu	2	0	LT-3
.......................uccacagcuuucuugaacuu	1	0	JCK-1
.......................uccacagcuuucuugaacuu	9	0	LCK-3
.......................uccacagcuuucuugaacuu	2	0	LT-1
.......................uccacagcuuucuugaacuu	7	0	LT-2
.......................uccacagcuuucuugaacuu	6	0	JCK-2
.......................uccacagcuuucuugaacuuu	4	0	LCK-3
.......................uccacagcuuucuugaacuuC	1	1	LT-2
.......................uccacagcuuucuugaacAuu	4	1	LT-2
.......................Cccacagcuuucuugaacuuu	1	1	JCK-1
.......................uccacagcuuucuugaacuuu	1	0	JCK-2
.......................uccacagcuuucuugaacuuu	5	0	LT-2
.......................uccacagcuuucuugaacuuu	2	0	LT-1
.......................uccacagcuuucuugaacuuu	2	0	JCK-1
.......................uccacagcuuucuugaacuuu	4	0	LT-3
.......................uccacagcuuucuugaacuuuU	3	1	LT-1
.......................uccacagcuuucuugaacuuuU	1	1	JCK-1
.......................uccacagcuuucuugaacuuuU	3	1	JCK-2
.......................uccacagcuuucuugaacuuuU	1	1	LT-2
.......................uccacagcuuucuugaacuuuc	2	0	LT-1
.......................uccacagcuuucuugaacuuuc	1	0	JCK-2
.......................uccacagcuuucuugaacuuuU	6	1	LCK-3
.......................uccacagcuuucuugaacuuuA	10	1	LCK-3
.......................uccacagcuuucuugaacuuuc	1	0	JCK-1
.......................uccacagcuuucuugaacuuuU	2	1	JCK-3
.......................uccacagcuuucuugaacuuuU	2	1	LT-3
.......................uccacagcuuucuugaacuuuA	1	1	LT-2
.......................uccacagcuuucuugaacuuuUu	2	1	LCK-3
........................ccacagcuuucuugaacC	2	1	JCK-1
........................ccacagcuuucuugaCcu	1	1	JT-1
........................ccacCgcuuucuugaacu	1	1	LT-3
........................ccacagcuuucuugaacG	1	1	LT-3
........................ccacagcuuucuugaacu	69	0	JCK-3
........................ccacagcuuucuugaacA	1	1	JCK-1
........................ccacagcuuucuugaacA	2	1	LT-2
........................ccacagcuuucuugaCcu	1	1	LT-3
........................ccacagcuuucuugaCcu	2	1	LCK-3
........................ccacagcuuucuugaacC	1	1	LT-3
........................ccCcagcuuucuugaacu	1	1	LT-2
........................ccacagcuuucuugaaUu	2	1	LCK-3

Mature / Star

```
                      Mature                                                     Star
uuugcaaguccuggucaugcuuuuccacagcuuucuugaacuuucuuucuuucuguuuagucucuuuuuuaagauugaacaccauugaaagcucaagaaagcugugggagaauauggcaaauuugggauuuuaauuuuuuuauuuuuguuuuuauuuuuuauuuuuccuucaacuacucuucagcugaugaugauguugcacacagaaagaaagaaagaaagaaagaaagagagaucucagaagcuaaau
........................ccacagcuuuAuugaacu................................................................................................................................................................................................    1    1    LT-3
........................ccacagcuuucuugaacu................................................................................................................................................................................................    51   0    JT-1
........................ccacagcuuucuugCacu................................................................................................................................................................................................    2    1    LT-3
........................ccacagcCuucuugaacu................................................................................................................................................................................................    1    1    LT-3
........................ccacagcuuucuugaacA................................................................................................................................................................................................    1    1    LT-3
........................ccacagcuuucuugaacu................................................................................................................................................................................................    51   0    JCK-1
........................ccacagcuuucuugaacu................................................................................................................................................................................................    342  0    LT-3
........................ccacagcuuucuugaacu................................................................................................................................................................................................    89   0    JCK-2
........................ccacagcuuucuugaacu................................................................................................................................................................................................    104  0    LT-1
........................ccacagcuuucuugaacA................................................................................................................................................................................................    1    1    JCK-2
........................ccacagcuuucuugaacu................................................................................................................................................................................................    24   0    LCK-1
........................ccacagcuuuUuugaacu................................................................................................................................................................................................    4    1    LT-3
........................ccacagcuuucuugaacu................................................................................................................................................................................................    14   0    JT-2
........................ccacagcuuucuugaacu................................................................................................................................................................................................    36   0    LCK-2
........................ccacagcuuucuugaaUu................................................................................................................................................................................................    2    1    LT-1
........................ccacagcuuucuugaaUu................................................................................................................................................................................................    5    1    LT-3
........................ccacagcuuucuugaacu................................................................................................................................................................................................    20   0    JT-3
........................Acacagcuuucuugaacu................................................................................................................................................................................................    1    1    LT-2
........................ccacagcuuucuugaacu................................................................................................................................................................................................    318  0    LCK-3
........................ccacagcuuucuugaacu................................................................................................................................................................................................    301  0    LT-2
........................ccacagcuuucuugaacuA...............................................................................................................................................................................................    10   1    JT-1
........................ccacagcuuucuugaacuA...............................................................................................................................................................................................    17   1    JCK-2
........................ccacagcuuucuugaacuu...............................................................................................................................................................................................    246  0    LT-2
........................ccacagcuuucuugaacuu...............................................................................................................................................................................................    252  0    JCK-2
........................ccacagcuuucuugaaUuu...............................................................................................................................................................................................    3    1    LT-1
........................ccacagcuuucuugaaAuu...............................................................................................................................................................................................    3    1    LT-3
........................ccacagcuuucuugCacuu...............................................................................................................................................................................................    1    1    JCK-2
........................ccacagcCuucuugaacuu...............................................................................................................................................................................................    3    1    LCK-3
........................ccacagcuuucuugaacuu...............................................................................................................................................................................................    68   0    JT-1
........................ccacagcuuucuugaCcuu...............................................................................................................................................................................................    1    1    LCK-3
........................ccacagcuuucuugaacAu...............................................................................................................................................................................................    1    1    JCK-1
........................ccacagcuuucuugaaUuu...............................................................................................................................................................................................    5    1    JCK-2
........................ccacagcuuucuugaacuu...............................................................................................................................................................................................    66   0    LT-1
........................ccacagcuuucuugaacuC...............................................................................................................................................................................................    3    1    LT-2
........................ccacagcuuucuugCacuu...............................................................................................................................................................................................    2    1    JCK-3
........................ccacagcuuucuugaacuC...............................................................................................................................................................................................    5    1    LT-1
........................ccacagcuuucuugaacCu...............................................................................................................................................................................................    1    1    LCK-3
........................ccacagcuuucuugaCcuu...............................................................................................................................................................................................    1    1    JT-2
........................cNacagcuuucuugaacuu...............................................................................................................................................................................................    1    1    JCK-2
........................ccacagcuuucuugaaUuu...............................................................................................................................................................................................    4    1    JCK-3
........................ccacagcuuucuugaacuu...............................................................................................................................................................................................    251  0    LT-3
........................ccacagcuuucuugaacuA...............................................................................................................................................................................................    19   1    LT-2
........................ccacCgcuuucuugaacuu...............................................................................................................................................................................................    1    1    JT-1
........................ccCcagcuuucuugaacuu...............................................................................................................................................................................................    1    1    LT-3
........................ccacagcuuucuugaacuA...............................................................................................................................................................................................    6    1    LT-1
........................ccacagcuuucuugaacuA...............................................................................................................................................................................................    3    1    JT-2
........................ccacagcuuucuugaaUuu...............................................................................................................................................................................................    3    1    JCK-1
........................ccacagcuuucuugaacuu...............................................................................................................................................................................................    168  0    JCK-1
........................ccacagcuuucuugaaUuu...............................................................................................................................................................................................    6    1    LCK-3
........................ccacagcuuucuugaacuu...............................................................................................................................................................................................    42   0    JT-3
........................ccacagcuuucNugaacuu...............................................................................................................................................................................................    1    1    JCK-1
........................cNacagcuuucuugaacuu...............................................................................................................................................................................................    1    1    JT-1
........................ccacagcuuucuugaCcuu...............................................................................................................................................................................................    1    1    LT-2
........................ccacagcuuucuugCacuu...............................................................................................................................................................................................    2    1    LT-2
........................ccacagcuuucuugaacuC...............................................................................................................................................................................................    12   1    JCK-2
........................ccacagcuuucuugaacuu...............................................................................................................................................................................................    349  0    LCK-3
........................ccacagcuuucuugaaUuu...............................................................................................................................................................................................    7    1    LT-3
........................ccacagcuuucuugaaAuu...............................................................................................................................................................................................    1    1    JCK-2
........................ccacagcuuucuugaacuA...............................................................................................................................................................................................    41   1    LCK-3
........................ccacagcuuucuugaacuC...............................................................................................................................................................................................    10   1    JT-1
........................ccacagcuuucuugaacuu...............................................................................................................................................................................................    34   0    JT-2
........................ccacagcuuucuugaacuA...............................................................................................................................................................................................    9    1    LT-3
........................ccacagcuuucuugaaUuu...............................................................................................................................................................................................    9    1    LT-2
........................ccacagcuuucuugaacuA...............................................................................................................................................................................................    11   1    JCK-1
........................ccacCgcuuucuugaacuu...............................................................................................................................................................................................    1    1    JCK-2
........................ccacagcuuucuugaacuu...............................................................................................................................................................................................    180  0    JCK-3
........................ccacagcuuucuugaacuC...............................................................................................................................................................................................    4    1    JCK-3
........................cNacagcuuucuugaacuu...............................................................................................................................................................................................    1    1    JCK-3
........................ccacagcuuucuugaacuA...............................................................................................................................................................................................    8    1    JCK-3
........................ccacagcuuucuugaacuC...............................................................................................................................................................................................    4    1    LT-3
```

Mature Star

uuugcaaguccuggucaugcuuuuccacagcuuucuugaacuuucuuucuuucuguuuagucucuuuuuuaagauugaacaccauugaaagcucaagaaagcugugggagaauauggcaaauuugggauuuuaauuuuuuuauuuuuguuuuuauuuuuuauuuuuccuucaacuacucuucagcugaugaugauguugcacacagaaagaaagaaagaaagaaagaaagagagaucucagaagcuaaau			
........................ccacagcuuucuugaacuA	5	1	JT-3
........................ccaAagcuuucuugaacuu	1	1	JCK-1
........................ccacagcuuucuugaacuC	2	1	JCK-1
........................ccaNagcuuucuugaacuu	1	1	JCK-1
........................ccacCgcuuucuugaacuu	2	1	LCK-3
........................ccacagcuuucuugaacuuC	18	1	JCK-3
........................ccacagcuuucuugaacAuu	5	1	LCK-3
........................ccacagcuuucuugaaUuuu	7	1	LT-3
........................ccacagcuuucuugaacuuu	21	0	JT-2
........................ccacaAcuuucuugaacuuu	1	1	JCK-3
........................ccacagcuuucuugCacuuu	1	1	LCK-3
........................ccacagcuuucuugaacuuC	20	1	LT-3
........................ccacagcuuucuuAaacuuu	1	1	LT-2
........................ccacagcuuucuugaacuCu	4	1	JCK-1
........................Ucacagcuuucuugaacuuu	4	1	JCK-2
........................ccacagcuuucuugaacuAu	9	1	JCK-2
........................ccaAagcuuucuugaacuuu	1	1	LCK-1
........................ccacagcuuucuugaacuuG	1	1	LT-1
........................ccacagcuuucuugaacuuA	8	1	JT-3
........................ccacCgcuuucuugaacuuu	1	1	JT-1
........................ccacagcuuucuugaaUuuu	13	1	LCK-3
........................ccacagcuuucuugaacuuu	43	0	JT-1
........................ccacagcuuucuugaacuuC	4	1	LT-1
........................ccacagcuuucuugCacuuu	1	1	JCK-2
........................ccacagcuuucuugaacuuC	24	1	JCK-2
........................ccacaNcuuucuugaacuuu	1	1	LCK-1
........................ccacagcuuucuugaaUuuu	18	1	LCK-2
........................ccNcagcuuucuugaacuuu	1	1	LCK-1
........................ccacagGuuucuugaacuuu	1	1	JT-3
........................ccacagcuuucuugaacuCu	7	1	JCK-3
........................ccacagcuuucuugaaUuuu	4	1	JCK-1
........................ccacagcuuucuugaacuuG	7	1	JCK-1
........................ccacagcuuucuugaacuAu	6	1	JCK-1
........................ccacagcuuucuugaacuCu	8	1	LT-3
........................ccacagcuuucuugaacuuu	439	0	LT-3
........................ccacagcuuucuugaacuuG	2	1	JCK-2
........................ccacagcuuucuugaacuuA	48	1	LT-3
........................ccacagcuuucuugaacGuu	1	1	JCK-1
........................ccacagcuuucuugaacuCu	4	1	LT-1
........................ccacagcuuucuugaacuuu	169	0	LT-1
........................ccacCgcuuucuugaacuuu	2	1	LT-2
........................ccacagcuuCcuugaacuuu	1	1	LT-3
........................ccacagcuuucuugNacuuu	1	1	LCK-1
........................ccacagcuuucuugaacuuu	77	0	LCK-1
........................ccacagcuuucuugaacuuu	506	0	LCK-3
........................ccacagcuuucuugaacAuu	1	1	JCK-2
........................ccacagcuuucuugaacuuu	31	0	JT-3
........................ccacagcuuucuugaacuuA	10	1	LT-1
........................ccacagcuuucuugaacuuu	71	0	LCK-2
........................cNacagcuuucuugaacuuu	1	1	JCK-1
........................Ucacagcuuucuugaacuuu	1	1	JCK-1
........................ccacagcuuucuugaacuuA	69	1	JCK-2
........................ccacagcuuucuugaacuCu	4	1	LT-2
........................ccacagcuuucuugaaAuuu	5	1	LCK-3
........................ccacagcuuucuugaacuuC	15	1	JCK-1
........................ccacagcuuucuugaacuuA	51	1	LT-2
........................ccacagcuuucuugaacAuu	2	1	LT-1
........................ccacagcuuucuugaacuuu	495	0	JCK-2
........................ccacagcuuucuugaacuuu	334	0	JCK-1
........................ccacagcuuucuugaaUuuu	3	1	JCK-3
........................ccacagcuuucuugaacuuG	1	1	LT-3
........................ccacagcuuucuugCacuuu	1	1	LT-2
........................ccacagcuuucuugaacuuu	485	0	LT-2
........................ccacagcuuucuugaacAuu	2	1	JCK-1
........................ccacagcuuucuugaGcuuu	1	1	LCK-2
........................ccacCgcuuucuugaacuuu	3	1	JCK-3
........................ccacagcuuucuugaacAuu	3	1	LT-2
........................ccacagcuuucuugaCcuuu	1	1	JCK-3
........................ccacagcuuucuugaacuuA	40	1	LCK-3
........................ccacagcuuucuugaacuAu	9	1	LCK-3

```
                      Mature                                                      Star
uuugcaaguccuggucaugcuuuuccacagcuuucuugaacuuucuuucuuucuguuuagucucuuuuuuaagauugaacaccauugaaagcucaagaaagcugugggagaauauggcaaauuugggauuuuaauuuuuuuauuuuuguuuuuauuuuuuauuuuuccuucaacuacucuucagcugaugaugauguugcacacagaaagaaagaaagaaagaaagaaagagagaucucagaagcuaaau
........................ccacagcuuucuugaacAuu     2    1  LT-3
........................ccacCgcuuucuugaacuuu     1    1  JCK-1
........................ccacagcuuucuugaacuCu     17   1  JCK-2
........................ccacagcuuucuugaacuuC     5    1  LCK-3
........................ccacagcuuucuugaacuuA     53   1  JCK-3
........................ccacagcuuucuugaaUuuu     27   1  LCK-1
........................ccacagcuuucuugaacuuG     1    1  JCK-3
........................ccacagcuuucuugaacuAu     5    1  JCK-3
........................ccacagcuuucuugaacuuC     14   1  LT-2
........................ccacagcuuucuugaacuuA     11   1  JT-2
........................ccacagcuuucuugaacuAu     16   1  LT-2
........................ccacagcuuucuugaacuuA     41   1  JCK-1
........................ccacagcuuucuugaacuAu     9    1  LT-3
........................ccacagcuuucuugaacuuu     349  0  JCK-3
........................ccacagcCuucuugaacuuu     1    1  JCK-3
........................ccacagcuuucuugaacuuuc    9    0  JCK-3
........................ccacagcuuucuugaacuuuU    64   1  LCK-2
........................ccacagcuuucuugaacuuAc    5    1  LCK-3
........................ccacagcuuucuugaacuuuA    33   1  LT-1
........................ccacagcuuucuugaacuuuc    16   0  LT-2
........................ccacagcuuucuugaacuuuA    109  1  LCK-3
........................ccacagcuuucuugaacuuuA    178  1  JCK-2
........................ccacagcuuucuugaacuuCc    1    1  JCK-1
........................ccacagcuuucuugaacuuuc    4    0  JCK-1
........................ccacagcuuucuugaacuuuG    8    1  JCK-2
........................ccacagcuuucuugaacuuuU    52   1  LCK-1
........................ccacagcuuucuugaacuuCc    1    1  LCK-3
........................ccacagcuuucuugaacuAuc    1    1  LT-3
........................ccacagcuuucuugaacuuuc    3    0  LT-1
........................ccacagcuuucuugaacuuuA    82   1  LT-3
........................ccacagcuuucuugaacuuuU    349  1  LCK-3
........................ccacagcuuucuugaaUuuuc    1    1  LT-2
........................ccacagcuuucuugaacuuuA    146  1  LT-2
........................ccacagcuuucuugaCcuuuc    1    1  LT-1
........................ccacagcuuucuugaacuuuG    17   1  LCK-3
........................ccacagcuuucuugaacuuuc    8    0  JCK-2
........................ccacagcuuucuugaacuuAc    1    1  LT-2
........................ccacagcuuucuugaacuuuA    150  1  JCK-1
........................ccacagcuuucuugaacuuuG    6    1  LT-1
........................ccacagcuuucuugaacuuuU    223  1  LT-3
........................ccacagcuuucuugaacuuuA    90   1  JCK-3
........................ccacagcuuucuugaacuuuG    10   1  JCK-3
........................ccacagcuuucuugaacuuuc    30   0  LCK-2
........................ccacagcuuucuugaacuuuU    246  1  LT-2
........................ccacagcuuucuugaacuuuG    14   1  LT-2
........................ccacagcuuucuugaacuuuG    6    1  LT-3
........................ccacagcuuucuugaacuuuU    42   1  JT-1
........................ccacagcuuucuugaacuAuc    7    1  LCK-3
........................ccacagcuuucuugaacuuAc    3    1  LT-3
........................ccacagcuuucuugaacuuuU    13   1  JT-3
........................ccacagcuuucuugaacuuuU    2    1  JT-2
........................ccacagcuuucuugaacuuuc    27   0  LCK-1
........................ccacagcuuucuugaacuuAc    1    1  JCK-2
........................ccacagcuuucuugaacuuuA    7    1  JT-1
........................ccacagcuuucuugaacuuuG    9    1  JCK-1
........................ccacagcuuucuugaacuuuU    138  1  JCK-1
........................ccacagcuuucuugaacuuuc    29   0  LCK-3
........................ccacagcuuucuugaacuuuU    87   1  LT-1
........................ccacagcuuucuugaacuuuU    184  1  JCK-2
........................ccacagcuuucuugaacuuuc    8    0  LT-3
........................ccacagcuuucuugaacuuuU    153  1  JCK-3
........................ccacagcuuucuugaacuuCc    1    1  LT-3
........................ccacagcuuucuugaacuuucG   1    1  JCK-3
........................ccacagcuuucuugaacuuuUu   38   1  JCK-3
........................ccacagcuuucuugaacuuAcu   1    1  LT-1
........................ccacagcuuucuugaacuuucC   2    1  LT-3
........................ccacagcuuucuugaacuuucu   3    0  LT-3
........................ccacagcuuucuugaacuuucA   3    1  JCK-3
........................ccacagcuuucuugaacuuAcu   2    1  LT-3
........................ccacagcuuucuugaacuuuUu   22   1  LCK-3
```

```
                      Mature                                                               Star
uuugcaaguccuggucaugcuuuuccacagcuuucuugaacuuucuuucuuucuguuuagucucuuuuuuaagauugaacaccauugaaagcucaagaaagcugugggagaauauggcaaauuugggauuuuaauuuuuuuauuuuuguuuuuauuuuuuauuuuuccuucaacuacucuucagcugaugaugauguugcacacagaaagaaagaaagaaagaaagaaagagagaucucagaagcuaaau
........................ccacagcuuucuugaacuuucu............................................................................................................................................................................................................   1   0   JCK-1
........................ccacagcuuucuugaacuuuAu............................................................................................................................................................................................................  16   1   LT-2
........................ccacagcuuucuugaacuuuUu............................................................................................................................................................................................................  29   1   JCK-1
........................ccacagcuuucuugaacuuuGu............................................................................................................................................................................................................   5   1   LCK-3
........................ccacagcuuucuugaacuuuAu............................................................................................................................................................................................................  13   1   LT-3
........................ccacagcuuucuugaacuuucu............................................................................................................................................................................................................   4   0   JCK-3
........................ccacagcuuucuugaacuuuUu............................................................................................................................................................................................................  25   1   LT-3
........................ccacagcuuucuugaacuuAcu............................................................................................................................................................................................................   1   1   JCK-1
........................ccacagcuuucuugaacuuuAu............................................................................................................................................................................................................  23   1   LCK-3
........................ccacagcuuucuugaacuuuUu............................................................................................................................................................................................................   4   1   JT-3
........................ccacagcuuucuugaacuAucu............................................................................................................................................................................................................   2   1   LCK-3
........................ccacagcuuucuugaacuuuAu............................................................................................................................................................................................................  23   1   JCK-2
........................ccacagcuuucuugaacuuuAu............................................................................................................................................................................................................   1   1   JT-3
........................ccacagcuuucuugaacuuAcu............................................................................................................................................................................................................   3   1   LT-2
........................ccacagcuuucuugaacuuucG............................................................................................................................................................................................................   5   1   JCK-2
........................ccacagcuuucuugaacuuucA............................................................................................................................................................................................................   4   1   LT-2
........................ccacagcuuucuugaacuuucu............................................................................................................................................................................................................   4   0   JCK-2
........................ccacagcuuucuugaacuuAcu............................................................................................................................................................................................................   4   1   JCK-3
........................ccacagcuuucuugaacuuAcu............................................................................................................................................................................................................   4   1   JCK-2
........................ccacagcuuucuugaacuuucA............................................................................................................................................................................................................   3   1   LT-3
........................ccacagcuuucuugaacuuuUu............................................................................................................................................................................................................  39   1   LT-2
........................ccacagcuuucuugaacuuuAu............................................................................................................................................................................................................  19   1   JCK-1
........................ccacagcuuucuugaacuuucA............................................................................................................................................................................................................   5   1   JCK-2
........................ccacagcuuucuugaacuuuAu............................................................................................................................................................................................................  16   1   JCK-3
........................ccacagcuuucuugaacuuuUu............................................................................................................................................................................................................   7   1   LT-1
........................ccacagcuuucuugaacuuuUu............................................................................................................................................................................................................   2   1   JT-2
........................ccacagcuuucuugaacuuuGu............................................................................................................................................................................................................   3   1   LT-1
........................ccacagcuuucuugaacuuucA............................................................................................................................................................................................................   1   1   JCK-1
........................ccacagcuuucuugaacuuucu............................................................................................................................................................................................................   1   0   LT-1
........................ccacagcuuucuugaacuuuAu............................................................................................................................................................................................................   4   1   LT-1
........................ccacagcuuucuugaacuuAcu............................................................................................................................................................................................................  23   1   LCK-3
........................ccacagcuuucuugaacuuuUu............................................................................................................................................................................................................  54   1   JCK-2
........................ccacagcuuucuugaacuuuAuu...........................................................................................................................................................................................................  13   1   LT-2
........................ccacagcuuucuugaacuuucuA...........................................................................................................................................................................................................   2   1   LCK-3
........................ccacagcuuucuugaacuuuAuu...........................................................................................................................................................................................................  25   1   LCK-3
........................ccacagcuuucuugaacuuuUuu...........................................................................................................................................................................................................   9   1   JCK-3
........................ccacagcuuucuugaacuuuAuu...........................................................................................................................................................................................................   3   1   LT-1
........................ccacagcuuucuugaacuuuAuu...........................................................................................................................................................................................................  14   1   JCK-1
........................ccacagcuuucuugaacuuuAuu...........................................................................................................................................................................................................  21   1   JCK-2
........................ccacagcuuucuugaacuuucAu...........................................................................................................................................................................................................   1   1   JCK-2
........................ccacagcuuucuugaacuuuUuu...........................................................................................................................................................................................................  14   1   LCK-3
........................ccacagcuuucuugaacuuucuA...........................................................................................................................................................................................................   1   1   LT-2
........................ccacagcuuucuugaacuuuGuu...........................................................................................................................................................................................................   1   1   JCK-2
........................ccacagcuuucuugaacuuuAuu...........................................................................................................................................................................................................   4   1   LT-3
........................ccacagcuuucuugaacuuucuu...........................................................................................................................................................................................................   1   0   JCK-3
........................ccacagcuuucuugaacuuucCu...........................................................................................................................................................................................................   1   1   JCK-2
........................ccacagcuuucuugaacuuuUuu...........................................................................................................................................................................................................  11   1   JCK-2
........................ccacagcuuucuugaacuuucAu...........................................................................................................................................................................................................   1   1   JCK-3
........................ccacagcuuucuugaacuuuAuu...........................................................................................................................................................................................................   4   1   JT-3
........................ccacagcuuucuugaacuuuUuu...........................................................................................................................................................................................................   4   1   JCK-1
........................ccacagcuuucuugaacuuuUuu...........................................................................................................................................................................................................   5   1   LT-2
........................ccacagcuuucuugaacuuuAuu...........................................................................................................................................................................................................   1   1   JT-2
........................ccacagcuuucuugaacuuuUuu...........................................................................................................................................................................................................   5   1   LT-1
........................ccacagcuuucuugaacuuuUuu...........................................................................................................................................................................................................   4   1   LT-3
........................ccacagcuuucuugaacuuuAuu...........................................................................................................................................................................................................  13   1   JCK-3
........................ccacagcuuucuugaacuuuUuuu..........................................................................................................................................................................................................   2   1   JCK-1
........................ccacagcuuucuugaacuuucuCu..........................................................................................................................................................................................................   1   1   JCK-2
........................ccacagcuuucuugaacuuuAuuu..........................................................................................................................................................................................................   2   1   JCK-2
........................ccacagcuuucuugaacuuuAuuu..........................................................................................................................................................................................................   3   1   JCK-1
........................ccacagcuuucuugaacuuucAuu..........................................................................................................................................................................................................   1   1   JCK-2
........................ccacagcuuucuugaacuuuAuuu..........................................................................................................................................................................................................   1   1   JCK-3
........................ccacagcuuucuugaacuuuUuuu..........................................................................................................................................................................................................   3   1   JCK-3
........................ccacagcuuucuugaacuuuAuuu..........................................................................................................................................................................................................   1   1   LT-2
........................ccacagcuuucuugaacuuuUuuu..........................................................................................................................................................................................................   1   1   LT-2
........................ccacagcuuucuugaacuuuAuuu..........................................................................................................................................................................................................   1   1   LT-3
........................ccacagcuuucuugaacuuuAuuu..........................................................................................................................................................................................................   1   1   LT-1
........................ccacagcuuucuugaacuuuUuuu..........................................................................................................................................................................................................   2   1   JCK-2
........................ccacagcuuucuugaacuuuUuuu..........................................................................................................................................................................................................   3   1   LT-3
.........................cacagcuuucuugaacuu...............................................................................................................................................................................................................   1   0   LT-3
.........................cacagcuuucuugaacuu...............................................................................................................................................................................................................   5   0   LCK-3
```

```
                      Mature                                                                     Star
uuugcaaguccuggucaugcuuuuccacagcuuucuugaacuuucuuucuuucuguuuagucucuuuuuuaagauugaacaccauugaaagcucaagaaagcugugggagaauauggcaaauuugggauuuuaauuuuuuuauuuuuguuuuuauuuuuuauuuuuccuucaacuacucuucagcugaugaugauguugcacacagaaagaaagaaagaaagaaagaaagagagaucucagaagcuaaau
.........................cCcagcuuucuugaacuu...............................................................................................................................................................................................   1   1   LCK-3
.........................cacagcuuucuugaacuu...............................................................................................................................................................................................   3   0   LT-2
.........................cacagcuuucuugaacuu...............................................................................................................................................................................................   3   0   JCK-3
.........................cacagcuuucuugaacuuu..............................................................................................................................................................................................   6   0   LT-3
.........................cacagcuuucuugaacuuu..............................................................................................................................................................................................   1   0   JCK-1
.........................cacagcuuucuugaacuuA..............................................................................................................................................................................................   2   1   JCK-3
.........................cacagcuuucuugaacuuA..............................................................................................................................................................................................   1   1   JCK-1
.........................cacagcuuucuugaacuAu..............................................................................................................................................................................................   2   1   LT-2
.........................cacagcuuucuugaacuuu..............................................................................................................................................................................................   10  0   JT-3
.........................cacagcuuucuugaacuuu..............................................................................................................................................................................................   8   0   JCK-2
.........................cacagcuuucuugaacuuu..............................................................................................................................................................................................   1   0   JT-2
.........................cacagcuuucuugaacuuu..............................................................................................................................................................................................   2   0   JCK-3
.........................cacagcuuucuugaacuuu..............................................................................................................................................................................................   4   0   LT-2
.........................cacagcuuucuugaacuuu..............................................................................................................................................................................................   2   0   LT-1
.........................cacagcuuucuugaacuuA..............................................................................................................................................................................................   1   1   LT-3
.........................cacagcuuucuugaacuuuU.............................................................................................................................................................................................   3   1   JCK-1
.........................cacagcuuucuugaacuuuU.............................................................................................................................................................................................   5   1   JCK-2
.........................cacagcuuucuugaacuuuU.............................................................................................................................................................................................   1   1   LT-1
.........................cacagcuuucuugaacuuuA.............................................................................................................................................................................................   1   1   JCK-3
.........................cacagcuuucuugaacuuuU.............................................................................................................................................................................................   5   1   JCK-3
.........................cacagcuuucuugaacuuuU.............................................................................................................................................................................................   2   1   LT-2
.........................cacagcuuucuugaacuuuA.............................................................................................................................................................................................   1   1   LT-3
.........................cacagcuuucuugaacuuuU.............................................................................................................................................................................................   5   1   LCK-3
.........................cacagcuuucuugaacuuuU.............................................................................................................................................................................................   2   1   LT-3
.........................cacagcuuucuugaacuuuA.............................................................................................................................................................................................   4   1   LCK-3
.........................cacagcuuucuugaacuuucu............................................................................................................................................................................................   2   0   JCK-2
.........................cacagcuuucuugaacuuucu............................................................................................................................................................................................   2   0   JCK-1
.........................cacagcuuucuugaacuuucu............................................................................................................................................................................................   9   0   JCK-3
.........................cacagcuuucuugaacuuucu............................................................................................................................................................................................   3   0   LT-1
.........................cacagcuuucuugaacuuuAuu...........................................................................................................................................................................................   8   1   JCK-2
.........................cacagcuuucuugaacuuuAuu...........................................................................................................................................................................................   3   1   JCK-1
.........................cacagcuuucuugaacuuuUuu...........................................................................................................................................................................................   1   1   LT-1
.........................cacagcuuucuugaacuuuUuu...........................................................................................................................................................................................   2   1   LT-3
.........................cacagcuuucuugaacuuuAuu...........................................................................................................................................................................................   2   1   JCK-3
.........................cacagcuuucuugaacuuAcuuu..........................................................................................................................................................................................   1   1   LT-1
..........................acagcuuucuugaacuuu..............................................................................................................................................................................................   1   0   LT-2
..........................acagcuuucuugaaUuuu..............................................................................................................................................................................................   6   1   LCK-3
..........................acagcuuucuugaacuuA..............................................................................................................................................................................................   2   1   LT-2
..........................acagcuuucuugaacuuuU.............................................................................................................................................................................................   3   1   LT-2
..........................acagcuuucuugaacuuuU.............................................................................................................................................................................................   1   1   LT-3
..........................acagcuuucuugaacuuuU.............................................................................................................................................................................................   4   1   LCK-3
..........................acagcuuucuugaacuuuA.............................................................................................................................................................................................   1   1   LT-1
..........................acagcuuucuugaacuuuUu............................................................................................................................................................................................   3   1   LT-3
.........................................uuucuGucuuucuguuua...............................................................................................................................................................................   1   1   LT-2
..........................................................................................gcucaagaaagcuguggg..............................................................................................................................................   1   0   JCK-2
..........................................................................................gcucaagaaagcuguggga.............................................................................................................................................   1   0   LT-3
..........................................................................................gcucaagaaagcugugggag............................................................................................................................................   3   0   JT-2
..........................................................................................gcucaagaaagcugugggag............................................................................................................................................   7   0   JT-3
..........................................................................................gcucaagaaagcugugggag............................................................................................................................................   2   0   JCK-1
..........................................................................................gcucaagaaagcugugggag............................................................................................................................................   2   0   JCK-3
..........................................................................................gcucaCgaaagcugugggag............................................................................................................................................   1   1   JT-3
..........................................................................................Acucaagaaagcugugggaga...........................................................................................................................................   1   1   JCK-1
..........................................................................................gcucaagaaagcugugggaga...........................................................................................................................................   13  0   JCK-1
..........................................................................................gcucaagaaagcugugggaga...........................................................................................................................................   17  0   JCK-3
..........................................................................................gcucaagaaagcugugggaga...........................................................................................................................................   1   0   LT-1
..........................................................................................gcucaagaaagcugugggaga...........................................................................................................................................   7   0   LCK-3
..........................................................................................gcucaagaaagcugugggaga...........................................................................................................................................   17  0   JCK-2
..........................................................................................gcucaagaaagcugugggaga...........................................................................................................................................   5   0   LT-3
...........................................................................................cucaagaaagcuguggga.............................................................................................................................................   2   0   JCK-3
...........................................................................................cucaagaaagcuguggga.............................................................................................................................................   1   0   JCK-2
...........................................................................................cucaagaaagcugugggag............................................................................................................................................   6   0   JT-2
...........................................................................................cucaagaaagcugugggag............................................................................................................................................   4   0   JCK-2
...........................................................................................cucaagaaagcugugggag............................................................................................................................................   2   0   JCK-1
...........................................................................................cucaagaaagcugugggag............................................................................................................................................   1   0   LT-3
...........................................................................................cucaagaaagcugugggag............................................................................................................................................   4   0   JT-3
...........................................................................................cucaagaaagcugugggag............................................................................................................................................   1   0   JCK-3
...........................................................................................cucaagaaagcugugggaga...........................................................................................................................................   23  0   LT-3
...........................................................................................cucaagaaagcugugggaga...........................................................................................................................................   8   0   JT-3
...........................................................................................cucaagaaagAugugggaga...........................................................................................................................................   1   1   JCK-2
...........................................................................................cucaagaaaAcugugggaga...........................................................................................................................................   1   1   LT-2
```

Mature uuccacagcuuucuugaacu — **Star** cucaagaaagcugugggaga

uuugcaaguccuggucaugcuuuuccacagcuuucuugaacuuucuuucuuucuguuuagucucuuuuuuaagauugaacaccauugaaagcucaagaaagcugugggagaauauggcaaauuugggauuuuaauuuuuuuauuuuuguuuuuauuuuuuauuuuuccuucaacuacucuucagcugaugaugauguugcacacagaaagaaagaaagaaagaaagaaagagagaucucagaagcuaaau

cucaagaaagcugugggaga	112	0	JCK-3
cucaagaaagcugugggaga	21	0	LT-2
cucaagaaagcugugggaga	6	0	JT-2
cucaagaaagcugugggaga	86	0	JCK-1
cucaCgaaagcugugggaga	1	1	JCK-2
cucaagaaagcugugggaga	19	0	LT-1
Uucaagaaagcugugggaga	1	1	JCK-2
cucaaUaaagcugugggaga	3	1	JCK-3
cucaagaaagcugugggagC	2	1	JCK-2
cNcaagaaagcugugggaga	2	1	JCK-3
cucaagaaagcugugggaga	111	0	JCK-2
cucaagaaagcugugggagC	2	1	JCK-3
cucaagaaagcugugggagaC	2	1	JCK-2
cucaagaaagcugugggagaU	1	1	JCK-3
cucaagaaagcugugggagaC	1	1	JCK-1
cucaagaaagcugugggagaUu	1	1	LT-3
ucaagaaagcugugggaga	1	0	JCK-1
ucaagaaagcugugggaga	1	0	JCK-3
ucaagaaagcugugggaga	3	0	JCK-2
aagaaagcugugggagaauauggc	2	0	JT-3
aagaaagcugugggagaauauggc	1	0	LT-3
aagaaagcugugggagaauauggc	1	0	LT-1
aagaaagcugugggagaauauggc	4	0	JT-2
aagaaagcugugggagaauauggc	10	0	JCK-2
aagaaagcugugggagaauauggc	4	0	JCK-1
aagaaagcugugggagaauauggc	10	0	JCK-3
aaagcugugggagaauauggc	1	0	JCK-3
aaagcugugggagaauauggc	2	0	JCK-2
aagcugugggagaauauggc	1	0	JCK-2
aagcugugggagaauauggc	3	0	LT-2
aagcugugggagaauauggc	1	0	JCK-3
aagcugugggagaauauggc	3	0	LT-1
aagcugugggagaauauggca	8	0	JT-2
aagcugugggagaauauggca	5	0	JT-3
aagcugugggagaauauggca	6	0	JCK-2
aagcugugggagaauauggca	3	0	LT-3
agcugugggagaauauggc	3	0	JCK-2
ugugggagaauauggcaaauu	1	0	JCK-3
ugugggagaauauggcaaauu	1	0	JCK-2
